# Modeling circadian variability of core-clock and clock-controlled genes in four tissues of the rat

**DOI:** 10.1371/journal.pone.0197534

**Published:** 2018-06-12

**Authors:** Panteleimon D. Mavroudis, Debra C. DuBois, Richard R. Almon, William J. Jusko

**Affiliations:** 1 Department of Pharmaceutical Sciences, School of Pharmacy and Pharmaceutical Sciences, State University of New York at Buffalo, Buffalo, NY, United States of America; 2 Department of Biological Sciences, State University of New York at Buffalo, Buffalo, NY, United States of America; University of Texas Southwestern Medical Center, UNITED STATES

## Abstract

Circadian clocks, present in almost all cells of the body, are entrained to rhythmic changes in the environment (e.g. light/dark cycles). Genes responsible for this timekeeping are named core-clock genes, which through transcriptional feedback interactions mediated by transcription factor binding to Ebox/RRE/Dbox elements can generate oscillatory activity of their expression. By regulating the transcription of other clock-controlled genes (CCGs) circadian information is transmitted to tissue and organ levels. Recent studies have indicated that there is a considerable variability of clock-controlled gene expression between tissues both with respect to the circadian genes that are regulated and to their phase lags. In this work, a mathematical model was adapted to explore the dynamics of core-clock and clock-controlled genes measured in four tissues of the rat namely liver, muscle, adipose, and lung. The model efficiently described the synchronous rhythmicity of core-clock genes and further predicted that their phases are mainly regulated by *Per2* and *Cry1* transcriptional delays and *Rev-Erba* and *Cry1* degradation rates. Similarly, after mining databases for potential Ebox/RRE/Dbox elements in the promoter region of clock-controlled genes, the phase variabilities of the same genes between different tissues were described. The analysis suggests that inter-tissue circadian variability of the same clock-controlled genes is an inherent component of homeostatic function and may arise due to different transcription factor activities on Ebox/RRE/Dbox elements.

## Introduction

To adapt to daily environmental changes and optimize energy utilization, organisms have evolved to maintain circadian rhythmicity in numerous biological processes such as sleep/wake cycles, rest/activity rhythms, and fasting/feedings cycles [1]. The mammalian circadian timing system retains a hierarchical organization where at the top of the hierarchy are pacemakers that are entrained by environmental cues such as light/dark cycles and subsequently orchestrate molecular clocks in most peripheral cell types through hormonal, neuronal or indirect cues [2]. The robust functioning of this circadian system as well as its efficient synchronization with the environmental rhythms is critical for the organism’s well-being. Its disruption has been linked with depression [3], obesity [4], diabetes [5], and cancer [6–10].

The molecular basis for generation of a circadian rhythm includes a group of genes called core-clock genes that, through transcriptional feedback interactions, can maintain rhythmic activity of their mRNA and protein levels [11]. In particular, CLOCK and BMAL proteins after heterodimerizing and translocating to the nucleus, regulate transcription of target genes containing Ebox sequences in their regulatory element including *Period* (*Per1*, *Per2*, *Per3*) and *Cryptochrome* (*Cry1* and *Cry2*). The PER and CRY proteins eventually translocate into the nucleus as a heterocomplex and inhibit their own transcription by preventing CLOCK:BMAL binding to Ebox elements. In the positive feedback loop, the Ebox-driven protein REV-ERBα inhibits transcription of *Bmal1* via ROR-elements (RREs) while DBP induces expression of genes by binding to Dbox elements in their regulatory region. Overall, this group of interconnected feedback loops shape a robust cellular oscillator that regulates these core-clock genes to express rhythmic activity [12–15]. One of the main current assumptions is that the phases of this small group of core-clock genes are entrained to environmental cycles through systemic signals such as circadian rhythmicity of cortisol and ultimately transmit this information at the tissue level through regulation of clock-controlled genes phases mainly by Ebox/RRE/and Dbox mediated transcription [16].

Numerous studies have found that circadian regulation is highly tissue specific [17–19]. Since circadian gene expression in tissues is affected by multiple systemic and tissue-specific signals, this tissue specificity can result from various factors. For instance, tissue entrainment by circadian changes of hormones such as melatonin and cortisol, rhythmic autonomic control, and indirect cues such as body temperature or feeding/fasting cycles may differentially regulate the expression of different genes [20–22]. Overall, by employing tissue-specific transcription factors, systemic signals can differentially influence gene expression in different body tissues [11]. Inter-tissue circadian variablity can be manifested in two ways. Either the genes maintaining circadian rhythmicity are significantly different in various tissues, or there are genes that are commonly oscillating in different tissues but retain different phases and amplitudes. This variability appears to be an essential characteristic of homeostasis and well-being [23]. Interestingly, recent reports have raised the hypothesis that circadian rhythmicity in each tissue is optimized in anticipation of the needs of the forthcoming light or dark phase [24]. Consequently, elucidating the mechanism that gives rise to the observed variabilities is of importance both to understand the underlying physiology and also indicates optimal ways of intervention and treatment.

Several mathematical models have been used to describe the network of core-clock and clock-controlled genes, shedding light on specific characteristics of the circadian network [12, 20, 25–29]. Recently, the interrelated system of negative and positive feedback loops among core-clock genes was described by a system of delay differential equations (DDEs) merging poorly characterized steps such as complex formation and localization into explicit delays [13]. Importantly, related efforts have considered regulation of clock-controlled genes by core-clock proteins binding to Ebox/RRE/and Dbox elements in the promoter region of target genes [14]. These modeling efforts provide a valuable mathematical test-bed allowing for further investigation of whether Ebox/RRE/Dbox interactions can explain inter-tissue variability of circadian genes, as well as the relative differences of these interactions in different tissues.

In this work, the mechanistic underpinnings resulting in the inter-tissue circadian variability observed in our studies involving Affymetrix gene array data from liver, muscle, adipose, and lung of Wistar rats was explored [19, 30–33]. In particular, by utilizing the model of [14] we were able to describe the synchronous rhythms of core-clock genes in the four rat tissues. Local sensitivity analysis of the core-clock gene network further showed that *Per2* and *Cry1* transcription delays together with *Cry1*and *Rev-Erba* degradation rates most strongly impact the phases of all genes involved in the network. After incorporating online database knowledge regarding the existence of Ebox/RRE/Dbox elements in the promoter regions of clock-controlled genes, the model well described the experimentally observed inter-tissue phase variabilities suggesting that these result from different post-translational regulations affecting Ebox/RRE/Dbox mediated transcription.

## Materials and methods

### Mathematical model

#### Core-clock genes

The model of core-clock genes describes the expression of 5 main genes namely *Bmal1*, *Rev-Erba*, *Per2*, *Cry1* and *Dbp* (Eqs 1–5). Each gene expression is modulated by transcription factors that bind to a specific regulatory element such as Ebox, RRE and Dbox. In particular, BMAL1 protein after heterodimerizing with CLOCK and translocating to the nucleus, induces the expression of core-clock genes retaining an Ebox element in their promoter region such as *Rev-Erba* (Eq 2), *Per2* (Eq 3), *Cry1* (Eq 4), and *Dbp* (Eq 5). PER and CRY proteins further repress Ebox mediated transcription not only of their own genes (Eqs 3 and 4) but also of *Rev-Erba* (Eq 2) and *Dbp* (Eq 5). Regarding Ebox-mediated transcription, recent efforts have indicated that both BMAL1-mediated activation as well as the two distinct inhibition mechanisms by PER2 and CRY1 are important for forward regulation [34, 35]. Next, REV-ERBα proteins repress the expression of genes that retain ROR/REV-ERB binding elements (RRE) in their promoter region such as *Bmal1* (Eq 1) and *Cry1* (Eq 4). Finally, DBP binds to Dbox elements, affecting expression of *Rev-Erba* (Eq 2), *Per2* (Eq 3), and *Cry1* (Eq 4). Entrainment by light/dark cycles is not explicitly taken into consideration in the model as it is implicitly incorporated in the fitted model parameters.

$$\frac{dBmal1}{dt}={\left(\frac{1}{1+\frac{{Rev-Erba}_{t-\tau_{Reverba}}}{ar1}}\right)}^{2}-d_{Bmal1}*Bmal1$$ (1)

$$\frac{dRev-Erba}{dt}={\left(\frac{1+b2*\frac{{Bmal1}_{t-\tau_{Bmal1}}}{ba2}}{1+\frac{{Bmal1}_{t-\tau_{Bmal1}}}{ba2}}\right)}^{3}*{\left(\frac{1}{1+\frac{{Per2}_{t-\tau_{Per2}}}{cr2}}\right)}^{3}*{\left(\frac{1}{1+\frac{{Cry1}_{t-\tau_{Cry1}}}{gr2}}\right)}^{3}*\left(\frac{1+f2*\frac{{Dbp}_{t-\tau_{Dbp}}}{fa2}}{1+\frac{{Dbp}_{t-\tau_{Dbp}}}{fa2}}\right)-d_{Rev-Erba}*Rev-Erba$$ (2)

$$\frac{dPer2}{dt}={\left(\frac{1+b3*\frac{{Bmal1}_{t-\tau_{Bmal1}}}{ba3}}{1+\frac{{Bmal1}_{t-\tau_{Bmal1}}}{ba3}}\right)}^{2}*{\left(\frac{1}{1+\frac{{Per2}_{t-\tau_{Per2}}}{cr3}}\right)}^{2}*{\left(\frac{1}{1+\frac{{Cry1}_{t-\tau_{Cry1}}}{gr3}}\right)}^{2}*\left(\frac{1+f3*\frac{{Dbp}_{t-\tau_{Dbp}}}{fa3}}{1+\frac{{Dbp}_{t-\tau_{Dbp}}}{fa3}}\right)-d_{Per2}*Per2$$ (3)

$$\frac{dCry1}{dt}={\left(\frac{1+b4*\frac{{Bmal1}_{t-\tau_{Bmal1}}}{ba4}}{1+\frac{{Bmal1}_{t-\tau_{Bmal1}}}{ba4}}\right)}^{2}*{\left(\frac{1}{1+\frac{{Per2}_{t-\tau_{Per2}}}{cr4}}\right)}^{2}*{\left(\frac{1}{1+\frac{{Cry1}_{t-\tau_{Cry1}}}{gr4}}\right)}^{2}*{\left(\frac{1}{1+\frac{{Rev-Erba}_{t-\tau_{Rev-Erba}}}{ar4}}\right)}^{2}*\left(\frac{1+f4*\frac{{Dbp}_{t-\tau_{Dbp}}}{fa4}}{1+\frac{{Dbp}_{t-\tau_{Dbp}}}{fa4}}\right)-d_{Cry1}*Cry1$$ (4)

$$\frac{dDbp}{dt}={\left(\frac{1+b5*\frac{{Bmal1}_{t-\tau_{Bmal1}}}{ba5}}{1+\frac{{Bmal1}_{t-\tau_{Bmal1}}}{ba5}}\right)}^{3}*{\left(\frac{1}{1+\frac{{Per2}_{t-\tau_{Per2}}}{cr5}}\right)}^{3}*{\left(\frac{1}{1+\frac{{Cry1}_{t-\tau_{Cry1}}}{gr5}}\right)}^{3}-d_{Dbp}*Dbp$$ (5)

The coefficients and rate constants describing the system are listed in Table 1. In Eqs 1–5, the mathematical form representing the induction or inhibition by a certain transcription factor is based on statistical mechanics and denotes the probability that RNA polymerase will bind to the promoter of interest in presence of an activator or repressor respectively [36]. Furthermore, the exponents in each induction or inhibition term represent the number of functional Ebox, RRE, or DBP elements in the regulatory region of each gene.

**Table 1:** Parameter values used for the simulation of the different rat tissue core-clock gene array data (Eqs 1–5). Transcriptional delays (τ_i_) and degradation rates (d_i_) were varied using literature-based values in order to simulate the observed data. Other parameters were set constant to their original values [14]. The All-Tissues parameters were calculated when gene array data from all tissues (liver, muscle, adipose, lung) were used in parameter estimation.

	Liver	Muscle	Adipose	Lung	All-tissues	Units	Description
**τ**_**Bmal1**_	4.89 [2.74, 7.05]	4.03 [3.64, 4.42]	7.41 [5.07, 9.75]	7.63 [7.61, 7.65]	6.28 [4.82, 7.75]	hr	Transcriptional delay of Bmal1
**τ**_**Rev-Εrba**_	0.66 [0.32, 1.01]	1.79 [1.57, 2]	0.55 [0.53, 0.57]	0.51 [0.51, 0.51]	0.56 [0.47, 0.64]	hr	Transcriptional delay of Rev-Erba
**τ**_**Per2**_	4.00 [3.08, 4.92]	3.89 [3.68, 4.11]	3.83 [2.91, 4.75]	3.98 [3.98, 3.98]	3.95 [3.35, 4.56]	hr	Transcriptional delay of Per2
**τ**_**Cry1**_	4.00 [3.64, 4.36]	3.08 [2.76, 3.41]	3.48 [2.92, 4.03]	3.16 [3.11, 3.2]	3.84 [3.46, 4.22]	hr	Transcriptional delay of Cry1
**τ**_**Dbp**_	3.00 [1.94, 4.06]	2.35 [2.16, 2.53]	2.20 [0.7, 3.69]	2.46 [2.44, 2.47]	3.00 [2.28, 3.72]	hr	Transcriptional delay of Dbp
**d**_**Bmal1**_	0.58 [0.21, 0.94]	0.37 [0.31, 0.43]	0.35 [0.26, 0.44]	0.37 [0.35, 0.39]	0.39 [0.3, 0.49]	hr^-1^	Degradation rate of Bmal1
**d**_**Rev-Εrba**_	0.61 [0.32, 0.9]	0.69 [0.67, 0.7]	0.62 [0.39, 0.85]	0.55 [0.54, 0.56]	0.58 [0.46, 0.7]	hr^-1^	Degradation rate of Rev-Erba
**d**_**Per2**_	0.24 [0.19, 0.3]	0.35 [0.33, 0.38]	0.32 [0.28, 0.36]	0.29 [0.29, 0.3]	0.27 [0.24, 0.3]	hr^-1^	Degradation rate of Per2
**d**_**Cry1**_	0.18 [0.12, 0.24]	0.21 [0.18, 0.23]	0.19 [0.14, 0.25]	0.20 [0.2, 0.2]	0.22 [0.17, 0.27]	hr^-1^	Degradation rate of Cry1
**d**_**Dbp**_	0.63 [0.454, 0.81]	0.49 [0.39, 0.59]	0.38 [0.32, 0.44]	0.48 [0.47, 0.49]	0.42 [0.38, 0.46]	hr^-1^	Degradation rate of Dbp
**ar1**	4.05					Concentration	Dissociation constant of REV-ERBa and its operator sequence on Bmal1 promoter
**ar4**	1.1					Concentration	Dissociation constant of REV-ERBa and its operator sequence on Cry1 promoter
**cr2**	1.83					Concentration	Dissociation constant of PER and its operator sequence on Rev-Erba promoter
**cr3**	33.5					Concentration	Dissociation constant of PER and its operator sequence on Per2 promoter
**cr4**	6.63					Concentration	Dissociation constant of PER and its operator sequence on Cry1 promoter
**cr5**	0.99					Concentration	Dissociation constant of PER and its operator sequence on Dbp promoter
**gr2**	80.2					Concentration	Dissociation constant of CRY and its operator sequence on Rev-Erba promoter
**gr3**	0.37					Concentration	Dissociation constant of CRY and its operator sequence on Per2 promoter
**gr4**	0.51					Concentration	Dissociation constant of CRY and its operator sequence on Cry1 promoter
**gr5**	1.02					Concentration	Dissociation constant of CRY and its operator sequence on Dbp promoter
**b2**	3.26						Fold change of Rev-Erba transcription by binding of BMAL1 to Ebox
**ba2**	0.51					Concentration	Dissociation constant of BMAL and its operator sequence on Rev-Erba promoter
**b3**	3.69						Fold change of Per2 transcription by binding of BMAL1 to Ebox
**ba3**	14.78					Concentration	Dissociation constant of BMAL and its operator sequence on Per2 promoter
**b4**	1.35						Fold change of Cry1 transcription by binding of BMAL1 to Ebox
**ba4**	1.06					Concentration	Dissociation constant of BMAL and its operator sequence on Cry1 promoter
**b5**	12.87						Fold change of Dbp transcription by binding of BMAL1 to Ebox
**ba5**	0.01					Concentration	Dissociation constant of BMAL and its operator sequence on Dbp promoter
**fa2**	0.19					Concentration	Dissociation constant of DBP and its operator sequence on Rev-Erba promoter
**f2**	1.23						Fold change of Rev-Erba transcription by binding of DBP to Dbox
**fa3**	0.58					Concentration	Effective equilibrium dissociation constant of DBP and its operator sequence on Per2 promoter
**f3**	11.69						Fold change of Per2 transcription by binding of DBP to Dbox
**fa4**	1.61					Concentration	Dissociation constant of DBP and its operator sequence on Cry1 promoter
**f4**	32.2						Fold change of Cry1 transcription by binding of DBP to Dbox

Values in square brackets indicate 95% confidence intervals.

In order to account for intermediate steps between a certain gene expression and its forward regulatory action in the promoter of a target gene (e.g. complex formation, nuclear localization), the equations were solved by considering constant delays based on literature values. As such, each gene "*i*" influences other gene transcriptions after a certain time delay *τ*_*i*_ that represents the translation, translocation into the nucleus, complex formation, and DNA binding of the gene. In accordance with [14], an underlying assumption is that these delays can be represented by literature values for the times between the gene "*i*" mRNA peak and the peak of its protein expression.

#### Clock-controlled genes (CCGs)

Clock-controlled genes are regulated by core-clock transcription factors binding to Ebox, RRE, and Dbox elements in their promoter region. The mathematical formulation follows the same framework as the core-clock genes and its general form is:

$$\frac{dCCGs}{dt}={\left(\frac{1+b*\frac{Bmal1_{t-\tau_{Bmal1}}}{ba}}{1+\frac{Bmal1_{t-\tau_{Bmal1}}}{ba}}\right)}^{n1}*{\left(\frac{1}{1+\frac{Per2_{t-\tau_{Per2}}}{cr}}\right)}^{n1}*{\left(\frac{1}{1+\frac{Cry1_{t-\tau_{Cry1}}}{gr}}\right)}^{n1}*{\left(\frac{1}{1+\frac{Rev-Erba_{t-\tau_{Rev-Erba}}}{ar}}\right)}^{n2}*{\left(\frac{1+f*\frac{Dbp_{t-\tau_{Dbp}}}{fa}}{1+\frac{Dbp_{t-\tau_{Dbp}}}{fa}}\right)}^{n3}-d*CCGs$$ (6)

**Fig 1:**
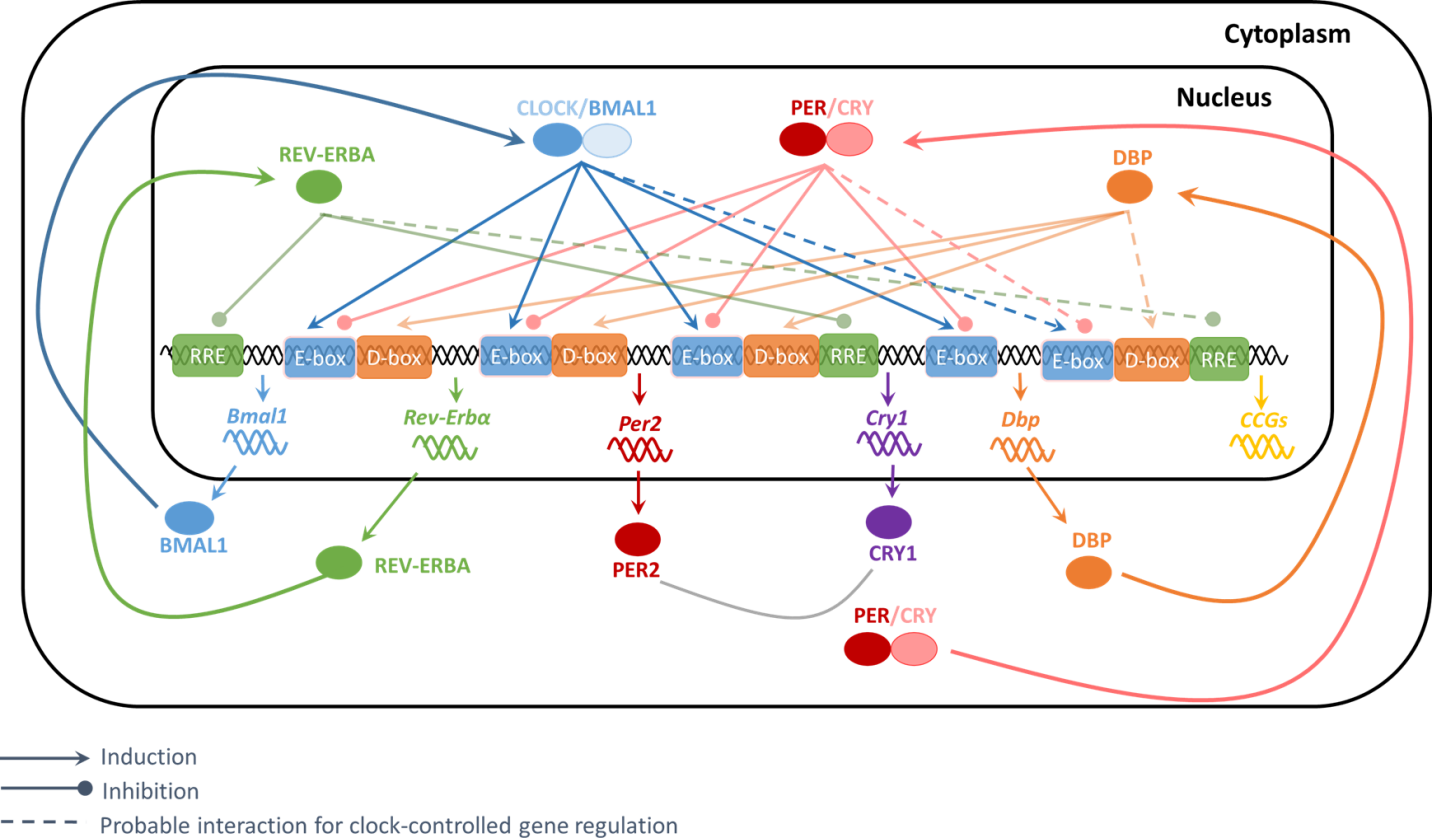
Schematic of the model. After heterodimerizing and translocating to the nucleus, CLOCK/BMAL1 induces the expression of target genes retaining an Ebox at their promoter (e.g. *Rev-Erba*, *Per2*, *Cry1*, *Dbp*). The PER/CRY heterocomplexes further inhibit this CLOCK/BMAL1 driven transcription. The REV-ERBa and DBP conclude the core-clock gene network by inhibiting or inducing genes that retain either an RRE or a DBP complex in their promoter regions. Clock-controlled genes (CCGs) are further regulated by core-clock transcription factors through binding to the respective Ebox, RRE, or Dbox elements at the promoter of the target gene.

Depending on the gene of interest and the number of Ebox, RRE, or Dbox elements in its promoter region, the respective exponents of Eq 6 are fixed to this value. If there are no Ebox, RRE, or Dbox elements the exponent is set to zero with the term equaling one. The investigation of each gene promoter region for potential Ebox, RRE, or Dbox elements is described in the section “*In-silico* promoter analysis”. All simulations were performed using Matlab R2016b dde23 solver for delay differential equations with constant delays. A schematic framework of the model is shown in Fig 1.

In order to evaluate the overall transcriptional regulation of a certain CCG via Ebox, RRE, and Dbox regulatory elements at a certain tissue *Ti*, the following variables within one cycle were calculated:

$${BMAL1}_{RegFac,Ti}={\left(\frac{1+b*\frac{{Bmal1}_{t-\tau_{Bmal1}}}{ba}}{1+\frac{{Bmal1}_{t-\tau_{Bmal1}}}{ba}}\right)}^{n1}$$ (7)

$${PER2}_{RegFac,Ti}={\left(\frac{1}{1+\frac{{Per2}_{t-\tau_{Per2}}}{cr}}\right)}^{n1}$$ (8)

$${CRY1}_{RegFac,Ti}={\left(\frac{1}{1+\frac{{Cry1}_{t-\tau_{Cry1}}}{gr}}\right)}^{n1}$$ (9)

$${REVERBA}_{RegFac,Ti}={\left(\frac{1}{1+\frac{{Rev-Erba}_{t-\tau_{Rev-Erba}}}{ar}}\right)}^{n2}$$ (10)

$${DBP}_{RegFac,Ti}={\left(\frac{1+f*\frac{{Dbp}_{t-\tau_{Dbp}}}{fa}}{1+\frac{{Dbp}_{t-\tau_{Dbp}}}{fa}}\right)}^{n3}$$ (11)

where *BMAL1*,*PER2*,*CRY1*,*REVERBA*,*DBP*_*RegFac*,*Ti*_ are variables (Regulation Factors) introduced to quantify the extent of transcription regulation via BMAL1, PER2, CRY1, REVERBA elements at a certain tissue *Ti*. To evaluate the regulation factors that determine the expression of a certain clock-controlled gene, the coefficient of variation (CV) of regulation factors (Eqs 7–11) was then calculated. Lastly, in order to evaluate whether there is a certain correlation between the phase lag of a certain gene in two tissues and BMAL1, PER2, CRY1, REVERBA, and DBP regulation factor variation in its promoter, the % change of the CV of regulation factors were calculated:

$${{\Delta CV(BMAL1}_{RegFac,}}_{Ti-Tj})\;\%=\frac{{max(CV(BMAL1}_{RegFac,Ti-Tj}))-{min(CV(BMAL1}_{RegFac,Ti-Tj})}{{max(CV(BMAL1}_{RegFac,Ti-Tj}))}*100$$ (12)

$${{\Delta CV(PER2}_{RegFac,}}_{Ti-Tj})\;\%=\frac{{{max(CV(PER2}_{RegFac,Ti-Tj}))-min(CV(PER2}_{RegFac,Ti-Tj}))}{{max(CV(PER2}_{RegFac,Ti-Tj}))}*100$$ (13)

$${{\Delta CV(CRY1}_{RegFac,}}_{Ti-Tj})\;\%=\frac{{{max(CV(CRY1}_{RegFac,Ti-Tj}))-min(CV(CRY1}_{RegFac,Ti-Tj}))}{{max(CV(CRY1}_{RegFac,Ti-Tj}))}*100$$ (14)

$${{\Delta CV(REVERBA}_{RegFac,}}_{Ti-Tj})\;\%=\frac{{{max(CV(REVERBA}_{RegFac,Ti-Tj}))-min(CV(REVERBA}_{RegFac,Ti-Tj}))}{{max(CV(REVERBA}_{RegFac,Ti-Tj}))}*100$$ (15)

$${{\Delta CV(DBP}_{RegFac,}}_{Ti-Tj})\;\%=\frac{{{max(CV(DBP}_{RegFac,Ti-Tj}))-min(CV(DBP}_{RegFac,Ti-Tj}))}{{max(CV(DBP}_{RegFac,Ti-Tj}))}*100$$ (16)

where max (CV(BMAL1, PER2, CRY1, REVERBA,DBP _RegFac, Ti-Tj_)) is the maximum coefficient of variation of BMAL1, PER2, CRY1, REVERBA, DBP regulation factor (Eqs 7–11) among tissues *Ti*, *Tj* and min (CV(BMAL1, PER2, CRY1, DBP_RegFac, Ti-Tj_)) is the minimum. These variables were introduced in order to investigate whether the different oscillatory characteristics of a certain CCGs in two tissues (e.g. different phase of a certain gene in two tissues), can be traced back to the different transcription regulation via Ebox, RRE, or Dbox elements.

### Parameter estimation and calculation of confidence intervals

To explain core-clock genes expression in different rat tissues, transcriptional delays (τ_i_) and degradation rates (d_i_) (Eqs 1–5) were optimized based on the available microarray data. Parameter optimization was performed in Matlab R2016b^®^_,_ using non-linear least square solver “lsqnonlin” and setting the upper and lower bounds for each parameter equal to the ranges indicated by experiments of mRNA decay [37–39] for degradation rates, and protein measurements [40–43] for transcriptional delays. For 95% confidence interval calculations, Matlab R2016b^®^ function “nlparci” was employed that utilizes the best estimates, residuals, and the Jacobian matrix of “lsqnonlin” least squares, in order to estimate the Wald (or normal) confidence intervals. The 95% confidence interval of a parameter p is given by:

$$\hat{p}\pm tinv(0.975,df)∙\sqrt{diag(v)}$$ (17)

where $\hat{p}$ is the optimal parameter value resulting from least squares, *t(0*.*975*,*df)* the student’s t inverse cumulative distribution function for 95% probability, *df* degrees of freedom (number of data–number of parameters), and *diag(v)* is the diagonal of the coefficient variance matrix calculated as:

$$v={\left(J^{T}J\right)}^{-1}∙\sigma^{2}$$ (18)

where J is the Jacobian matrix resulting from least squares, exponent T represents the transpose matrix, and *σ*^2^ the variance of the residuals. The variance of the residual *σ*^2^ is calculated as:

$$\sigma^{2}=\frac{norm(r)}{df}$$ (19)

where norm() the Euclidean norm, and r the residuals. The model parameters are listed in Table 1. For transcriptional delays and degradation rates, five values are shown that represent the values maintained for fitting the data from four different tissues as well as fitting all-tissues data together. The remaining parameters utilized values reported previously [14].

### *In-silico* promoter analysis

Binding of transcription factors to transcription factor binding sites (TFBSs) is key to transcriptional regulation [44, 45]. In this work, an *in-silico* search for Ebox, RRE, and Dbox elements was performed for regions 10 kb upstream and 5 kb downstream of the transcription start site (TSS) of the studied genes similar to the work of [46]. The transcription factors used for the identification of binding regions were BMAL1 (ARNTL) for Ebox, ROR for RRE, and DBP for Dbox. The corresponding position weight matrices (PWMs) for each transcription factor were mined from JASPAR2014 [47] and JASPAR2016 [48] databases and are shown in S2–S4 Figs. Regarding the genes of interest, their promoter sequence and gene annotations were adopted from UCSC Genome Browser [49, 50], and rat version (rn5) was used. In order to evaluate positive hits, an 85% profile score threshold was used. Data mining and forward computations of promoter analysis were performed in R using the Bioconductor package environment [51]. Output of our computations are shown in detail in Tables A-F in S1 Appendix.

### Sensitivity analysis

To evaluate the sensitivity of the core-clock gene phases, a local sensitivity analysis was performed. At each sensitivity analysis step, a transcriptional delay or degradation rate was varied by 10% of its nominal value while the other parameters were kept constant. Next, the sensitivity coefficients (s_i,j_) for the phases of the respective core-clock genes were calculated as:

$$s_{i,j}=\frac{\partial \phi_{i}}{\partial p_{j}}$$ (20)

where ∂ϕ_*i*_ is the phase difference of core-clock gene i, resulting from simulation with typical parameter values and simulation with 10% variation to parameter p_j_ and ∂*p_j_* is the difference between varied and nominal parameter values.

### Experimental data

The mathematical model for the core-clock and clock-controlled genes (Eqs 1–6) was validated and tested for its prediction power based on experiments performed in our lab. Detailed descriptions of the animal experiments are published [30–33]. Our research protocol adhered to the “Principles of Laboratory Animal Care” (National Institutes of Health Publication 85–23, revised 1985) and were approved by the State University of New York at Buffalo Institutional Animal Care and Use Committee. In brief, our studies involved 54 normal male Wistar rats from Harlan Laboratories (Indianapolis, IN) that were allowed to acclimatize in a constant 22°C environment equipped with a 12:12-h light-dark cycle with free access to standard rat chow and drinking water. Animals were sacrificed by exsanguination through the abdominal aorta on three successive days at 0.25, 1, 2, 4, 6, 8, 10, 11, and 11.75 h after lights on for the light period time points, and at 12.25, 13, 14, 16, 18, 20, 22, 23, and 23.75 h after lights on for time points in the dark period. Animals sacrificed at the same time on the three successive days were treated as triplicate measurements. Livers, gastrocnemius muscles, abdominal fat pads, and lungs were excised and frozen in liquid nitrogen immediately after sacrifice and stored at -80°C until RNA preparation. The biotinylated cRNAs from the tissue samples were hybridized to 54 individual Affymetrix GeneChips Rat Genome 230A for liver and muscle and 230A_2 for adipose and lung (Affymetrix, Santa Clara, CA). The data were submitted to Gene Expression Omnibus (GEO) (GSE8988 for liver, GSE8989 for muscle, GSE20635 for adipose, and GSE25612 for lung).

Affymetrix Microarray Suite 5.0 (Affymetrix) was used for initial data acquisition and analysis. The signal intensities were normalized for each chip with a distribution of all genes around the 50^th^-percentile for that chip. Using GeneSpring, the value of each probe set on each chip was normalized to the average of that probe set on all chips in that tissue set such that the expression pattern of all probe sets oscillated approximately around 1. In order to identify genes retaining circadian rhythmicity, the JTK_CYCLE non-parametric algorithm was employed [52]. Only the genes that retained a false discovery rate (FDR) lower than 0.1% were considered for mathematical analysis.

All core-clock genes of the model namely *Bmal1*, *Rev-Erba*, *Per2*, *Cry1* and *Dbp* were found to retain robust circadian oscillations in the four tissues. However, a *Cry1* probe set was not present in liver and muscle GeneChips (Affymetrix GeneChips Rat Genome 230A). For this reason, and due to the strong similarity of *Cry1* expression in adipose and lung with Zhang’s microarray data for the same tissues (GEO database GSE54650 [24]), *Cry1* expressions for liver and muscle were adopted from Zhang et al. after further considering phase and amplitude variabilities observed between the two experiments in liver and muscle (see S1 Fig).

## Results

**Fig 2:**
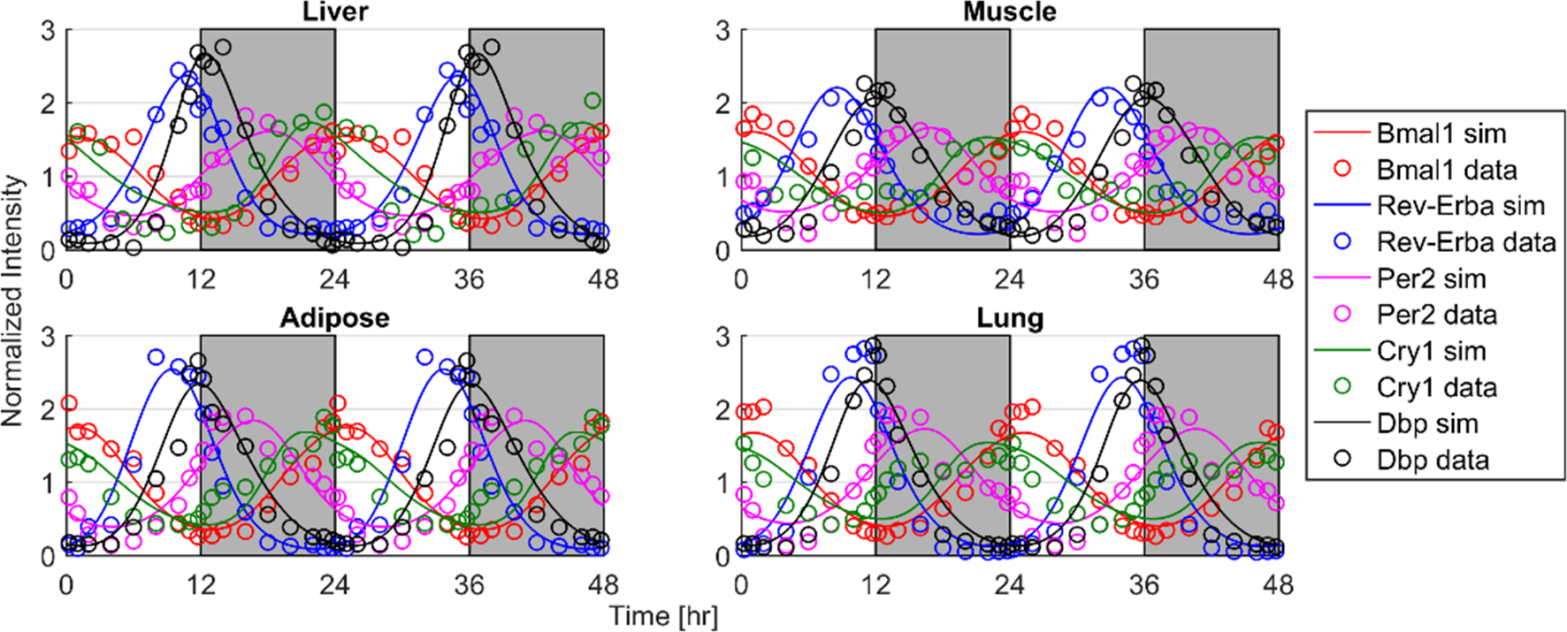
Model fittings (curves) of core-clock gene expression (mRNA—circles) in different rat’s tissues. Time of light/dark cycles are denoted by white/grey shading.

The first assessment was whether the expression of core-clock genes from different tissues could be described by the proposed mathematical model (Eqs 1–5). Fig 2 shows the model results for the core-clock genes *Bmal1*, *Rev-Erba*, *Per2*, *Cry1* in addition to the Affymetrix data from the different tissues. The model well characterizes the dynamics of the core-clock genes in the different rat tissues. Overall, they present high synchronicity in the different tissues. *Bmal1* maintains maximum expression in early morning, while *Rev-Erba* and *Dbp* peak at the transition of light/dark period. *Rev-Erba* expression for the case of muscle presents an earlier peak. *Per2* at all tissues peaks at the dark period followed by *Cry1* that peaks similar to *Bmal1* at late dark/early morning time.

**Fig 3:**
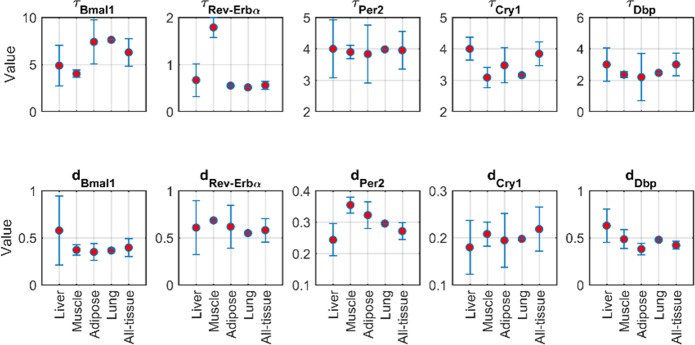
Transcriptional delay and degradation rate parameters estimated to describe the expression of core-clock genes for the different tissues. Error bars represent the 95% confidence interval. The y-axis provides the parameter values with definitions and units listed in Table 1. All-tissue depicts the parameter values resulting from fitting the data of all tissues concurrently (consensus model).

Fig 3 and Table 1 show the transcriptional delay and degradation rate parameters estimated in order to describe the core-clock gene expression in the four tissues. For most cases, transcriptional delays and degradation rates are similar among the different tissues as shown by the overlapping 95% confidence intervals of the different parameters (i.e. overlapping error bars). An exception is the transcriptional delay of *Rev-Erba* that for muscle retains a significantly higher value. However, due to the overall overlapping values of the delays and degradation rates, core-clock gene data from all tissues were fitted jointly (consensus model). The estimated delays and degradation rates of the consensus model are shown in Fig 3 as the All-tissue ordinate and in Table 1. Apart from the transcriptional delays and degradation rates, the remaining parameters of the model (Eqs 1–5) were set constant to the original values from [14].

**Fig 4:**
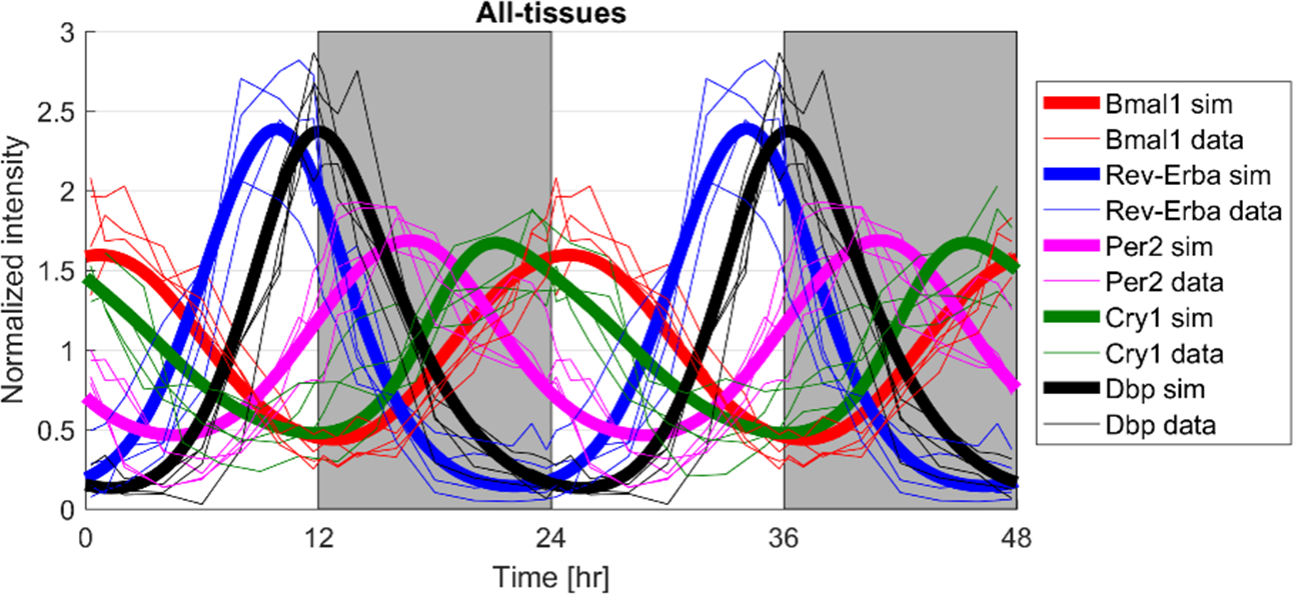
Responses of the consensus model describing the overall data from four tissues. Bold lines represent the joint model responses (sim) and thin lines the experimental data of core-clock genes in the different tissues. Light/dark periods are denoted by white/grey shading.

Since microarray data of core clock-genes in the different tissues present high synchronicity, Fig 4 shows the responses of a consensus model in which transcriptional delays and degradation rates were optimized based on data from all four tissues. The consensus model jointly describes the totality of the data very well capturing the amplitudes and the phases of core-clock genes in all tissues.

**Fig 5:**
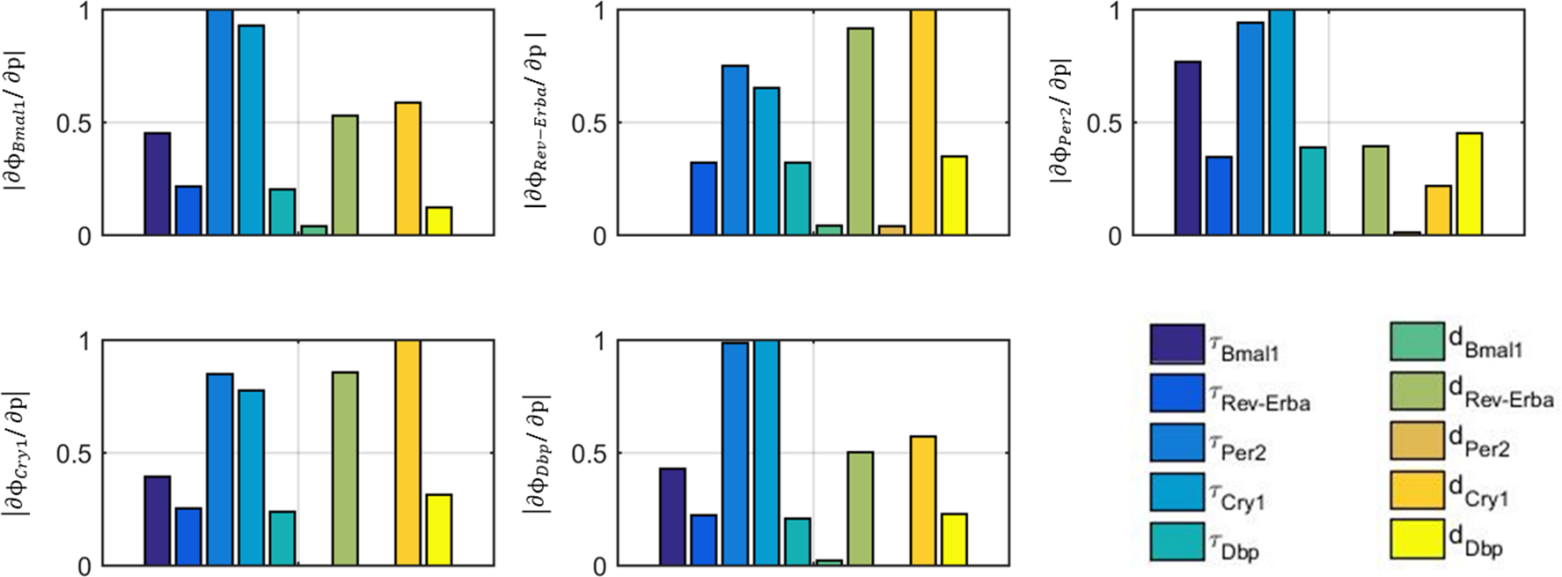
Local sensitivity analysis of the phases of core-clock genes upon changing transcriptional delays and degradation rates. Different subplots represent different sensitivity outputs that are the phases of the various core-clock genes (*Bmal1*, *Rev-Erba*, *Per2*, *Cry1*, *Dbp*). Bars indicate the sensitivity indices resulting by varying different parameters (transcriptional delays, degradation rates). The y-axis represents the absolute values of the normalized sensitivity coefficients (Eq 20).

The core-clock gene network retains a high level of interconnected negative and positive feedback loops that result in a strongly non-linear system. In order to explore how the variation of the different delays and degradation rates affect the phases of the core-clock genes, Fig 5 shows the results of a local sensitivity analysis. A gene phase is defined as the time of peak expression relative to 0 hr (time when lights are on) multiplied by 2π and divided by its period (expressed in radians). Among the transcriptional delays, the most sensitivite parameters were *Per2* and *Cry1* delays for regulating the phase of all core-clock genes. Regarding degradation rates, *Rev-Erba* and *Cry1* degradations highly affect the phase of *Bmal1*, *Rev-Erba*, *Cry1* and *Dbp* whereas degradation of *Per2* phase is also regulated by *Dbp* degradation.

**Fig 6:**
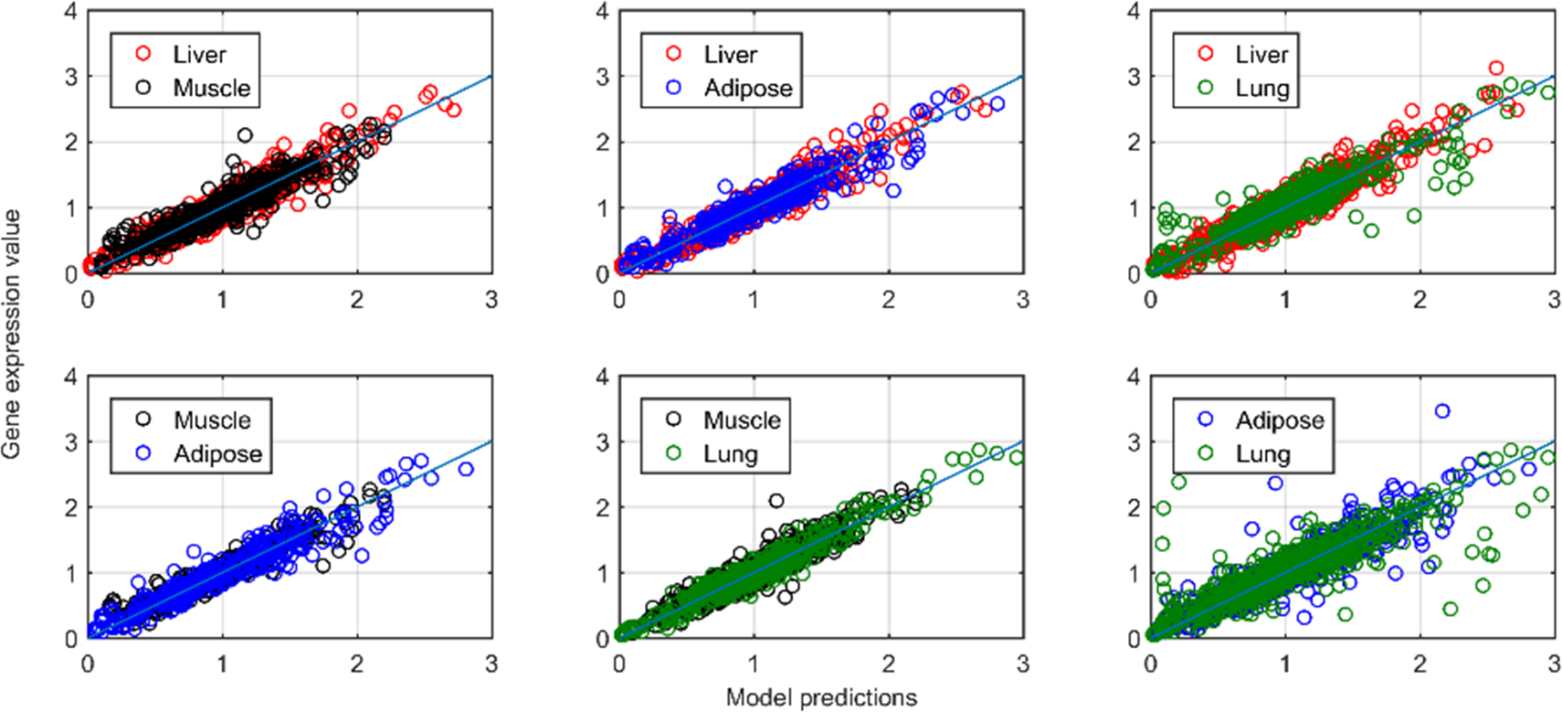
Model predictions relative to experimental data for clock-controlled genes oscillating jointly in pairs of rat tissues. The solid line depicts identity (y = x).

Based on the consensus model, the expression of clock-controlled genes that oscillate in combinations of two tissues were explored (Adipose/Lung, Liver/Adipose, Liver/Lung, Liver/Muscle, Muscle/Adipose, Muscle/Lung). Fig 6 shows model predictions relative to all experimental data over one cycle for all the common oscillating genes between each tissue pair. The available experimental data are well characterized as can be seen by the close proximity of simulations/data to the identity line. Detailed simulation profiles in addition to the microarray data are shown in S5–S10 Figs. For the case of common genes in adipose and lung, the dynamic of 1368247_at (S5 Fig) was not adequately described by the model resulting in a slightly larger deviation of prediction versus the data plot of Fig 6. In order to fit the experimental data, the dissociation constants (ba, cr, gr, ar, fa), fold transcription changes (b, f) and degradation rates (d) were estimated between the tissues.

**Fig 7:**
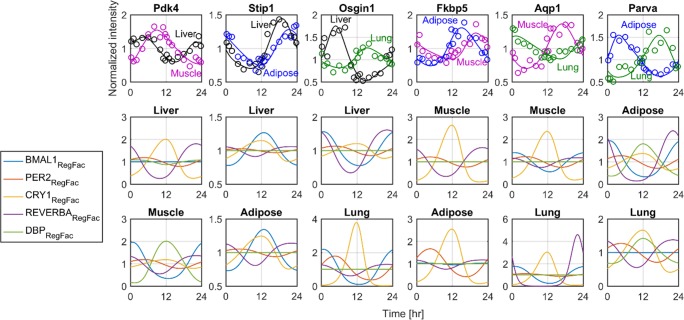
Ebox/RRE/Dbox regulation factor (RegFac) dynamics for the genes maintaining the highest phase difference among two tissues. Upper panel shows model simulations together with the experimental data for the genes that maintain the largest phase differences. For each subplot of the upper panel, the two lower panels indicate the regulation factor dynamics (BMAL1, PER2, CRY1, REVERBA, DBP) for the tissues shown in upper panel and indicated in the title. Regulation factors dynamics are normalized based on the mean values in order to better represent the factor that most clearly influences the clock-controlled gene expression.

In order to evaluate the underlying mechanism through which core-clock genes regulate clock-controlled genes phases, the clock-controlled genes with the largest phase lag among the different combination of tissues together with the Ebox/RRE/Dbox regulation factor dynamics are shown in Fig 7. The gene that shows the highest phase lag among liver and muscle was pyruvate dehydrogenase lipoamide kinase isozyme 4 (Pdk4). Regulation of Pdk4 in liver is mainly regulated by CRY1- and REVERBA-mediated inhibition, whereas muscle Pdk4 is mainly regulated by BMAL1- and DBP-mediated induction. Between liver and muscle the majority of clock-controlled genes oscillated in relative synchrony. Stress induced phosphoprotein 1 (*Sip1*) rhythmicity is mainly regulated by BMAL1 induction both in liver and muscle. For liver and lung, oxidative stress induced growth inhibitor 1 (*Osgin1*) had the largest phase lags; for liver its phase is mainly regulated by BMAL1 induction and lung by CRY1 inhibition. The FK506 binding protein 5 both in adipose and muscle is mainly regulated by CRY1 inhibition. Aquaporin 1 (*Aqp1*) phase is regulated mainly by CRY1 inhibition in muscle and REVERBA inhibition in lung. Lastly, Parvin a (*Parva*) phase in adipose is regulated by REVERBA inhibition whereas in lung by CRY1 inhibition.

**Fig 8:**
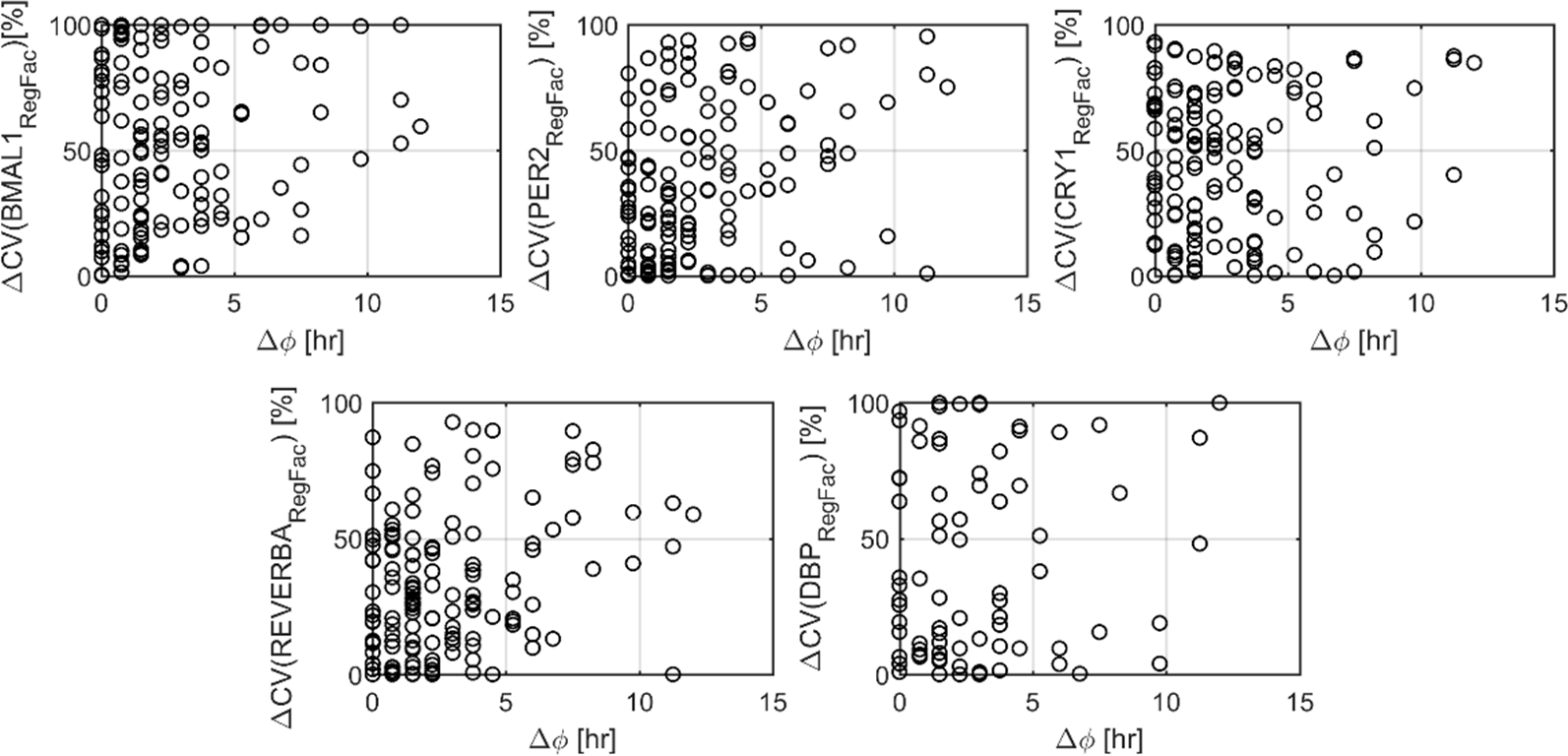
Relation between phase difference of the same gene in two tissues, and the variation of transcription regulation via BMAL1, PER2, CRY1, REVERBA, DBP mediated transcription (Eqs 12–16).

Lastly, in order to investigate whether there is a trend between phase difference and regulation factor activity, the relative differences of BMAL1, PER2, CRY1, REVERBA, DBP mediated transcription as quantified by Eqs 12–16 were compared between the same genes in two different tissues. Fig 8 shows the phase-difference of the same gene in two tissues versus the % change observed in BMAL1, PER2, CRY1, REVERBA, DBP regulation factors None of the investigated variations are correlated with the phase differences observed in gene oscillations. Among the five response elements, the one that shows the most consistent changes with respect to phase-lag appears to be REVERBA.

## Discussion

From genes to tissues, homeostasis reflects a temporal organization that allows for appropriate timing of physiological processes. Circadian rhythms represent an important indication of homeostasis and their dysfunction is often linked with multiple physiological disorders and ageing [53, 54]. Interestingly, recent experiments [19, 24] indicated that circadian variability, as exemplified by phase lags of individual genes between tissues, is an integral part of homeostasis. Our studies employed a mathematical model of core-clock and clock-controlled genes in order to investigate the potential mechanistic underpinnings that produce the observed circadian variability.

Two steps were followed. Initially, a model for core-clock genes (Eqs 1–5) was used to investigate the dynamics present in the liver, muscle, adipose and lung core-clock network. The model (Eqs 1–5) successfully fitted the core-clock gene expression data (Fig 2) efficiently capturing the phases, amplitudes, and periods. In order to describe inter-tissue variability, transcriptional delays (τ_i_) and degradation rates (d_i_) were optimized based on literature value boundaries [39–43]. The remaining parameters of Eqs 1–5 were set constant using values reported in [14]. The reason that only delays and degradation rates were varied was that there is prior knowledge regarding the physiological boundaries of these parameters. The rest of the parameters were found by global optimization techniques, as there is no evidence for their experimental values.

Based on protein measurements [40–43], transcriptional delays of core-clock genes could range up to 8 hours (e.g. *Bmal1* delay τ_Bmal1_ could range from 0 hours [40] to 8 hours [41]). Along the same lines, degradation rates have a considerable range of variation (e.g. degradation rate of *Per2* mRNA is among 0.24 and 0.8 h^-1^[37, 39]). In order to investigate how variations of transcriptional delays or degradation rates around their optimal value could impact the oscillatory characteristics of the core-clock genes and most importantly their phases, a local sensitivity analysis was enacted. Transcriptional delays and degradation rates were varied one at a time by 10% while the remaining parameters were kept constant. The phase changes were then evaluated and the absolute value of the sensitivity coefficients were plotted in Fig 5. Among the various transcriptional delays, sensitivity analysis indicated that *Per* and *Cry* delays most significantly affect the phase of all core-clock genes expression. PER and CRY proteins together with BMAL1 regulate transcription through Ebox elements. Given that Ebox is extensively present at the promoter region of almost all core-clock genes (*Rev-Erba*, *Per2*, *Cry1*, and *Dbp*), *Per* and *Cry* transcriptional delays high sensitivities are rather expected. Furthermore, sensitivity analysis indicates high sensitivities on certain degradation rates. In particular, *Rev-Erba* and *Cry1* degradation rates largely affect the phases of *Bmal1*, *Rev-Erba*, *Cry1*, and *Dbp* whereas degradation rate of *Dbp* affects phase of Per2. Overall, due to the high non-linearities of the core-clock gene network, parameter variations mediate their effects in an indirect way.

Recent experiments in mice and rats have shown that the genes maintaining circadian activity are significantly different among various tissues with a very small overlap [17–19, 24]. This suggests a highly tissue-specific clock that functions in order to optimize tissue-specific processes. Interestingly, in these studies, many of the genes that appear to oscillate in common are members of the core-clock gene network and present a relatively high synchronicity. Our analysis further confirms these observations, since core-clock genes in different tissues could be largely explained without statistically different transcriptional delays and degradation rates (Fig 3, overlapping 95% confidence intervals). Since our experiments also indicate a significant synchronicity of core-clock genes among tissues, a consensus fitting was applied in order to explain the totality of the core-clock gene data in the four tissues. The consensus model well characterized the core-clock gene data from all tissues (Fig 4). For each core-clock gene, the estimated transcriptional delays and degradation rates are similar to the averages when each tissue was fitted alone (Fig 3). In order to investigate the dynamics of clock-controlled genes (Eq 6), the transcriptional delays and degradation rates of the consensus model were used further.

Our measurements in rats showed that the majority of genes oscillating in combination of two or more tissues retain a relatively high synchronicity as measured by the phase difference of their peak mRNA expression [19]. Alongside, there are a considerable number of genes that maintain up to a 12-hour phase difference between their expressions from tissue to tissue. For example, neural epidermal growth factor-like 1 (*Nell1*) that plays a role in cell differentiation, peaks in lung at the light/dark transition whereas in adipose it peaks at the middle of the dark period 9 hours preceding lung’s peak. Similar to our work, Zhang et al [24] found that among 12 mice tissues examined, 1400 genes were phase-shifted with respect to themselves by at least 6 hours between two organs, with 131 genes completely antiphased. Although this circadian variability appears to be an integral part of homeostasis, currently there is no evidence of what mechanism can give rise to these dynamics. We assessed whether Ebox/RRE/Dbox mediated transcription of clock-controlled genes could result in the physiological variabilities observed in our prior experiments. The focus was only on those genes commonly oscillating in combination of two tissues. The underlying assumption is that the expression of the same gene in two tissues is regulated by the same systemic signals. Our analysis sheds light whether Ebox/RRE/Dbox mediated transcription can account for the observed inter-tissue circadian variability, assuming all other systemic signals are the same between tissues.

A critical step in investigating inter-tissue variabilities of clock-controlled gene expression is identifying the number of Ebox/RRE/Dbox elements in the promoter regions of the clock-controlled genes (Eq 6). Although there are many *in vitro* and *in vivo* experimental approaches to identify transcription factor binding sites (reviewed in [55]), similar to [46] such binding sites were recognized by scanning the position weight matrix (PWM) of the transcription factor (BMAL1, ROR, DBP) against the DNA of the promoter region of the corresponding clock-controlled gene (results are shown in Tables A-F in S1 Appendix as the n1,n2,n3 values). The absolute numbers of Ebox, RRE or Dbox elements that were found, were used as the n_1_, n_2_ and n_3_ exponents of Eq 6. Next, in order to describe the available experimental data, the respective parameters (i.e. b, ba, cr, f, fa, etc.) were optimized based on the data. Fig 6 shows the overall performance of our model in explaining clock-controlled gene expressions that oscillate in common in various tissue pairs. The totality of clock-controlled gene expression data were well explained. Since the number of Ebox, RRE and Dbox elements (n_1_, n_2_, n_3_ of Eq 6) in the promoter region of each clock-controlled gene were fixed based on mining of public databases, and the expression of core-clock genes (Eqs 1–5) were set constant to describe the all-tissue data (consensus model, Fig 4), the effective description of core-clock genes expression is not totally intuitive. This further indicates that inter-tissue variations of transcription factors dissociation constants may well account for the inter-tissue variabilities of clock-controlled gene phases. An example can be the ratio of reduced to oxidized NAD cofactors that strongly influence the CLOCK/BMAL1 binding activity and are often considered to be readouts of the cellular metabolic state [56].

By incorporating a bioinformatic approach to investigate Ebox/RRE/Dbox elements at the promoter region of the genes of interest, current work extends the model of Korencic et al [14] and enables its use so to investigate phase variabilities at the tissue level. In particular, using this model and our genome data for four tissues of rats [19, 30–33], we determined whether core-clock gene expression can efficiently account for the phases of clock-controlled genes and phase variabilities observed among the same genes in different tissues. Fig 7 shows the expression of genes that retain the highest phase lag among the different combination of tissue pairs along with the dynamics of the various regulation factors on their promoter region. Oxidative stress induced growth inhibitor 1 (*Osigi1*) is a gene that plays a role to the differentiation and proliferation of normal cells. In liver, *Osigi1* peaks at early light period (~5 hr) whereas in lung it peaks after 12 hr. For liver, *Osigi1* phase is regulated mainly by BMAL1 induction that occurs around 6 hours (τ_Bmal1_) and REVERBA inhibition that starts after 0.5 hour (τ_Rev-ERba_). On the other hand, *Osigi1* in lung is mainly driven by CRY1 inhibition. Similarly, aquaporin 1 (*Aqp1*) that translates for a water channel protein, maintains peak expression after 12 hr in muscle, when in lung retains a nadir. Again, regulation factor dynamics indicate the differential control of this gene expression that for muscle is mainly regulated by CRY1 inhibition whereas for lung by REVERBA inhibition. Overall, this analysis indicates that inter-tissue phase differences in gene expression are accompanied by different regulation in Ebox/RRE/Dbox elements. For the last step, we explored whether there is any specific correlation between phase lag of genes between two tissues, and the according % change of the coefficient of variation of Ebox, RRE, or Dbox mediated transcription. The results shown in Fig 8 do not point towards such a relationship. In particular, neither Ebox, RRE nor Dbox mediated transcription by itself can account for the phase lags observed. Based on the nonlinearities present in the clock gene network, as well as the overlap of interactions that can lead to the same phase (e.g. increase of RRE or decrease of Ebox binding), such as a result is expected.

In summary, this model of the clock network describes the dynamics of core-clock genes in four tissues of rats. Sensitivity analysis further indicates the major role of *Per2* and *Cry1* transcriptional delays as well as *Cry1*, *Rev-Erba* and *Dbp* degradation rates. The high synchronicity of core-clock genes in the four tissues, enabled the use of a consensus model that was expanded to account for Ebox/RRE/Dbox regulation of clock-controlled genes. The expression of all clock-controlled genes in the tissues tested as well as the incorporated variabilities were well described. This further indicates that phase differences of the same gene in two tissues are an integral part of homeostatic stability and can be sourced back to varying affinities of transcription factors in Ebox/RRE/Dbox transcription elements.

## Supporting information

S1 Fig *Cry 1* rhythms from Zhang et al. and Sukumaran et al. works. In our model for the case of liver and muscle (lower panel), data from the work of Zhang et al. were used [24] and further corrected based on the amplitude and phase differences found between our data [30–33] and these of [5] for the cases of adipose and lung (upper panel).
(TIF)

S2 Fig Position weight matrix of BMAL1. (TIF)

S3 Fig Position weight matrix of ROR. (TIF)

S4 Fig Position weight matrix of DBP. (TIF)

S5 Fig Simulated profiles of common probe sets oscillating in adipose and lung along with their respective data. (TIF)

S6 Fig Simulated profiles of common probe sets oscillating in liver and adipose along with their respective data. (TIF)

S7 Fig Simulated profiles of common probe sets oscillating in liver and lung along with their respective data. (TIF)

S8 Fig Simulated profiles of common probe sets oscillating in liver and muscle along with their respective data. (TIF)

S9 Fig Simulated profiles of common probe sets oscillating in muscle and adipose along with their respective data. (TIF)

S10 Fig Simulated profiles of common probe sets oscillating in muscle and lung along with their respective data. (TIF)

S1 Appendix Transcription factor analysis and data fitting result tables. (DOCX)
