# Supplementary figures and images for: Modeling circadian variability of core-clock and clock-controlled genes in four tissues of the rat
Source: PLoS One. 2018 Jun 12;13(6):e0197534. doi: 10.1371/journal.pone.0197534 (PMC5997360; doi:10.1371/journal.pone.0197534)

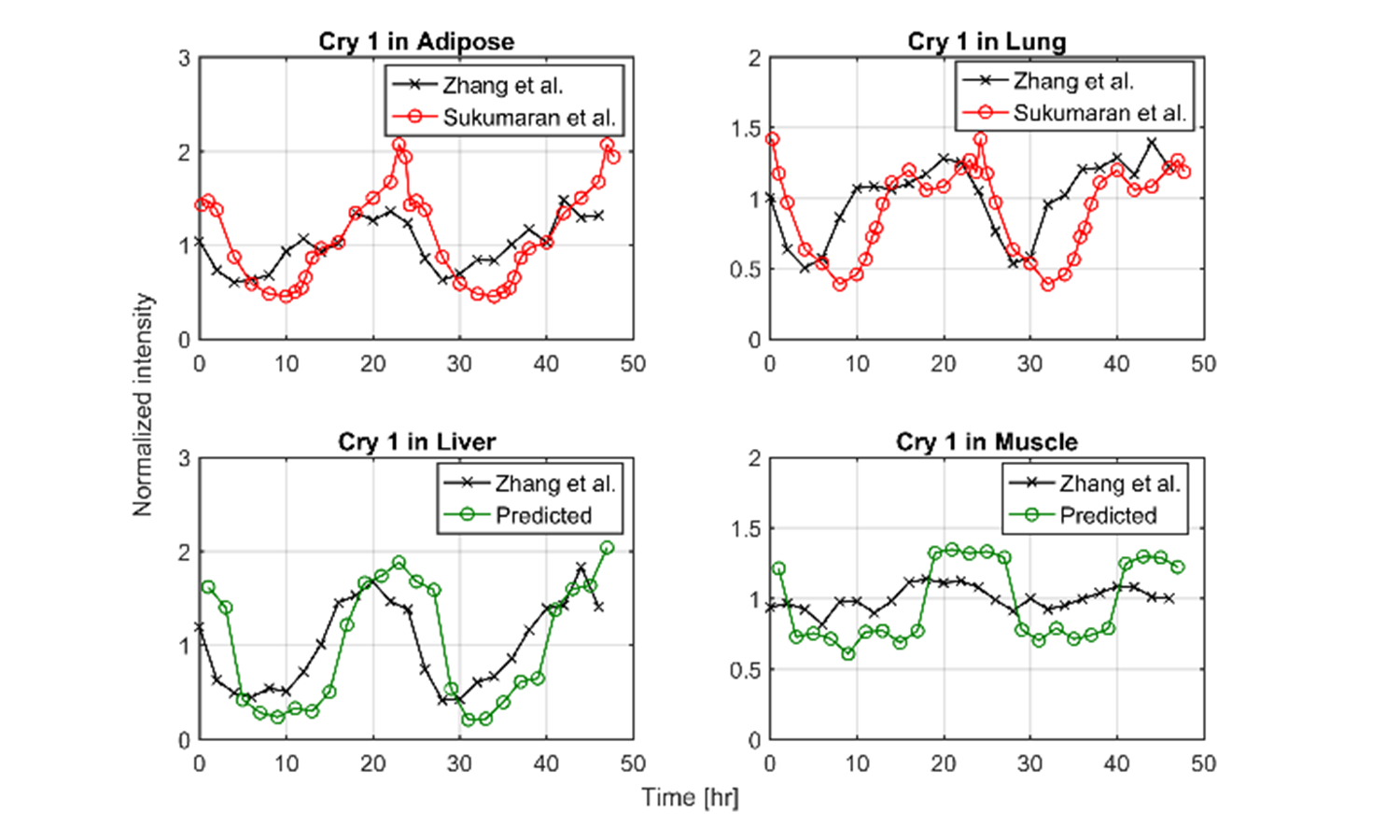

Supplement: S1 Fig — In our model for the case of liver and muscle (lower panel), data from the work of Zhang et al. were used [24] and further corrected based on the amplitude and phase differences found between our data [30–33] and these of [5] for the cases of adipose and lung (upper panel). (TIF) [file pone.0197534.s001.tif]

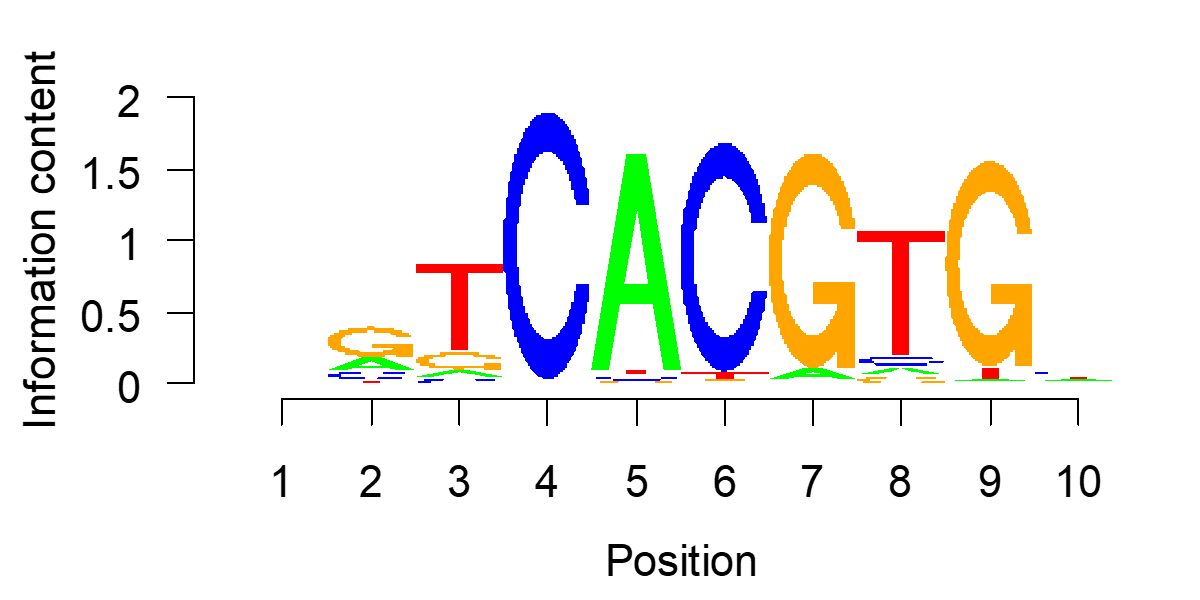

Supplement: S2 Fig — (TIF) [file pone.0197534.s002.tif]

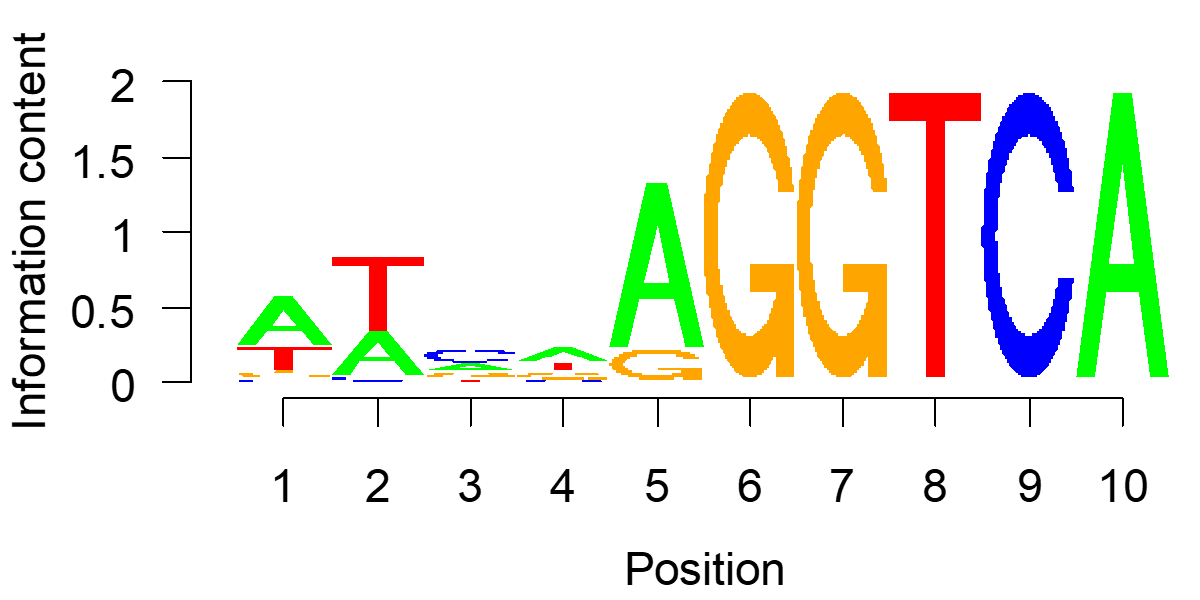

Supplement: S3 Fig — (TIF) [file pone.0197534.s003.tif]

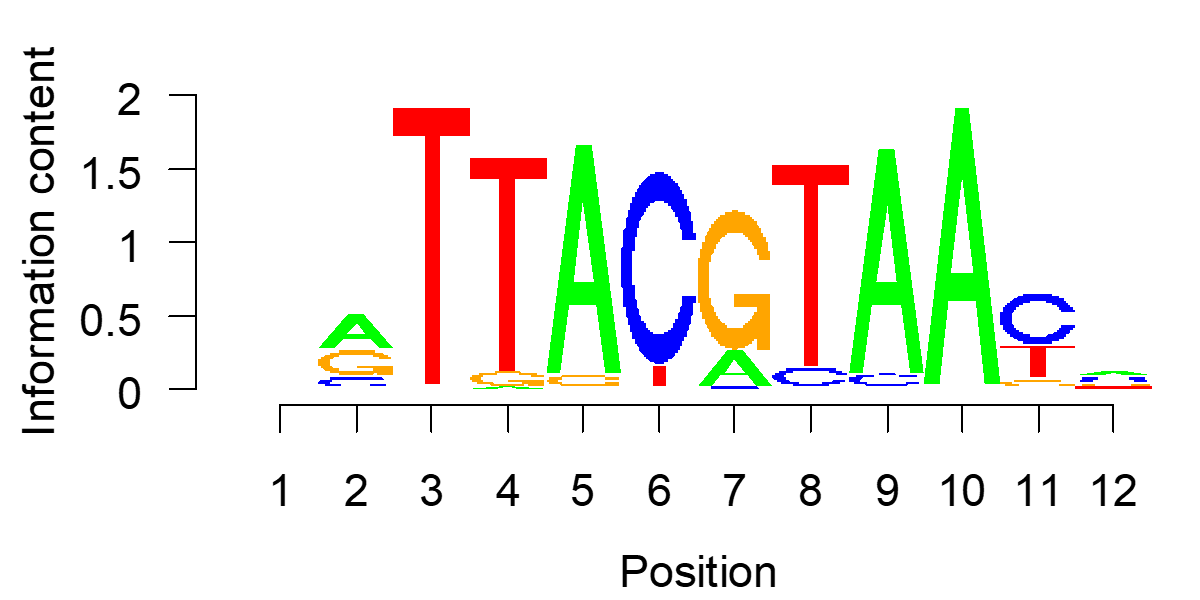

Supplement: S4 Fig — (TIF) [file pone.0197534.s004.tif]

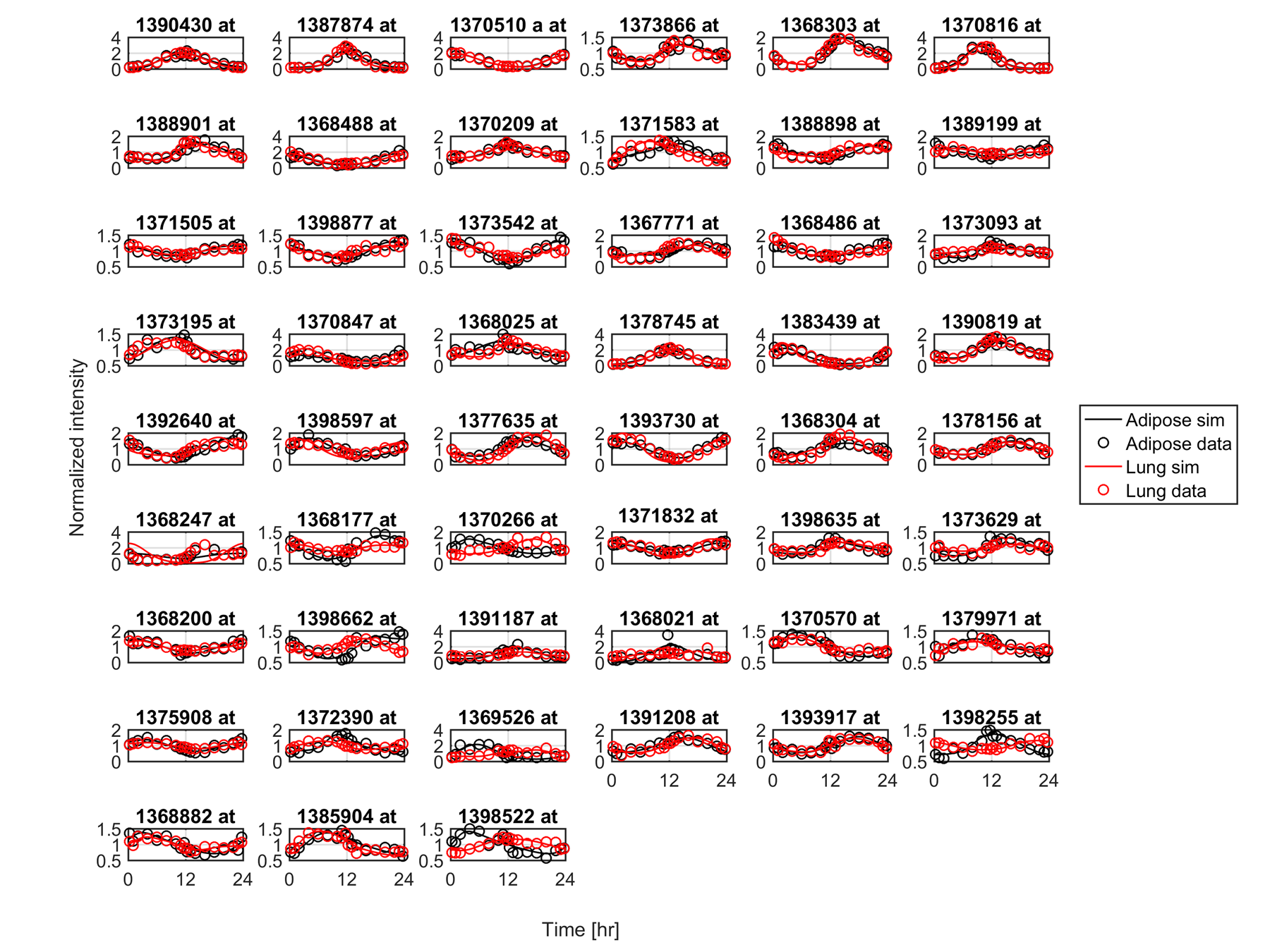

Supplement: S5 Fig — (TIF) [file pone.0197534.s005.tif]

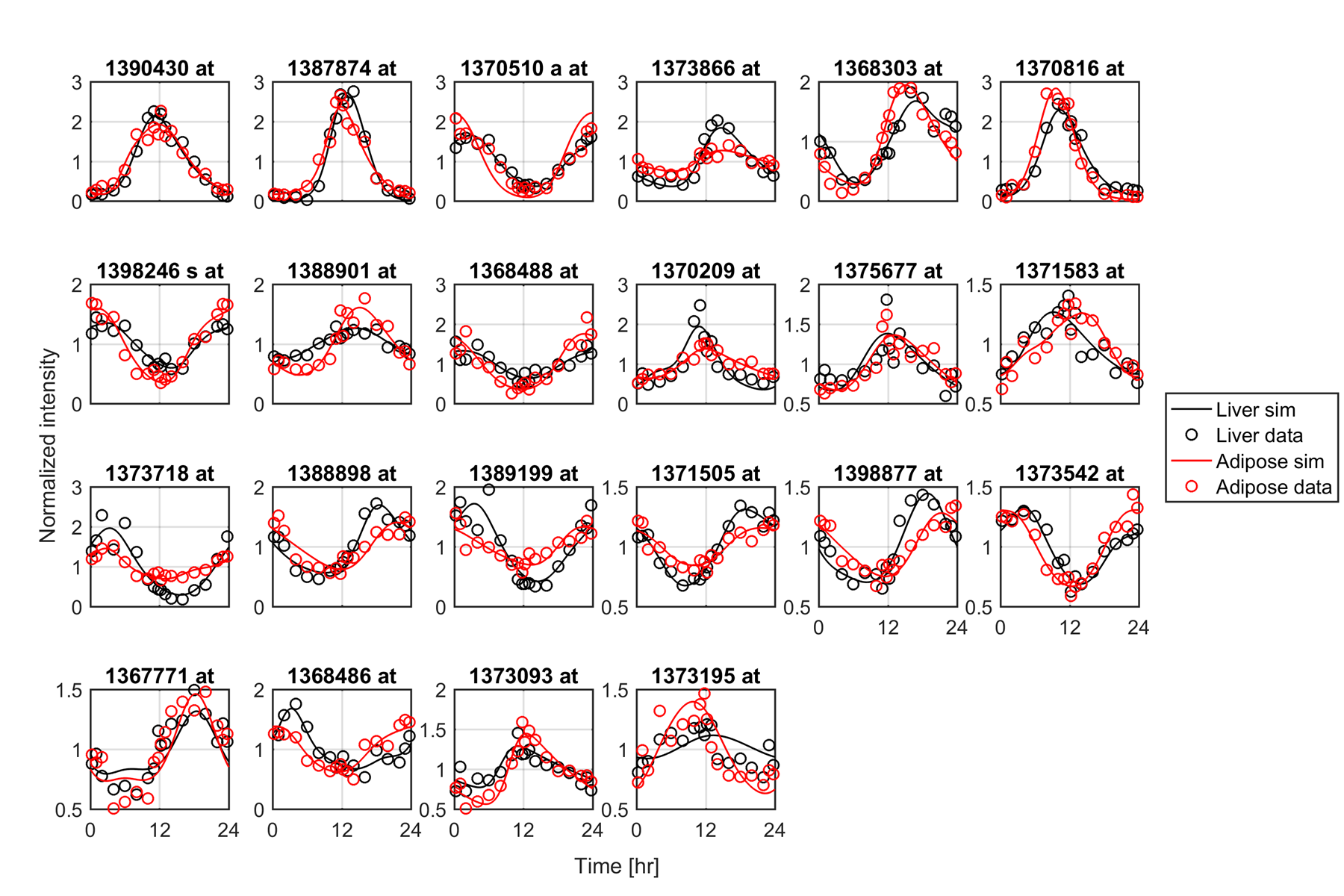

Supplement: S6 Fig — (TIF) [file pone.0197534.s006.tif]

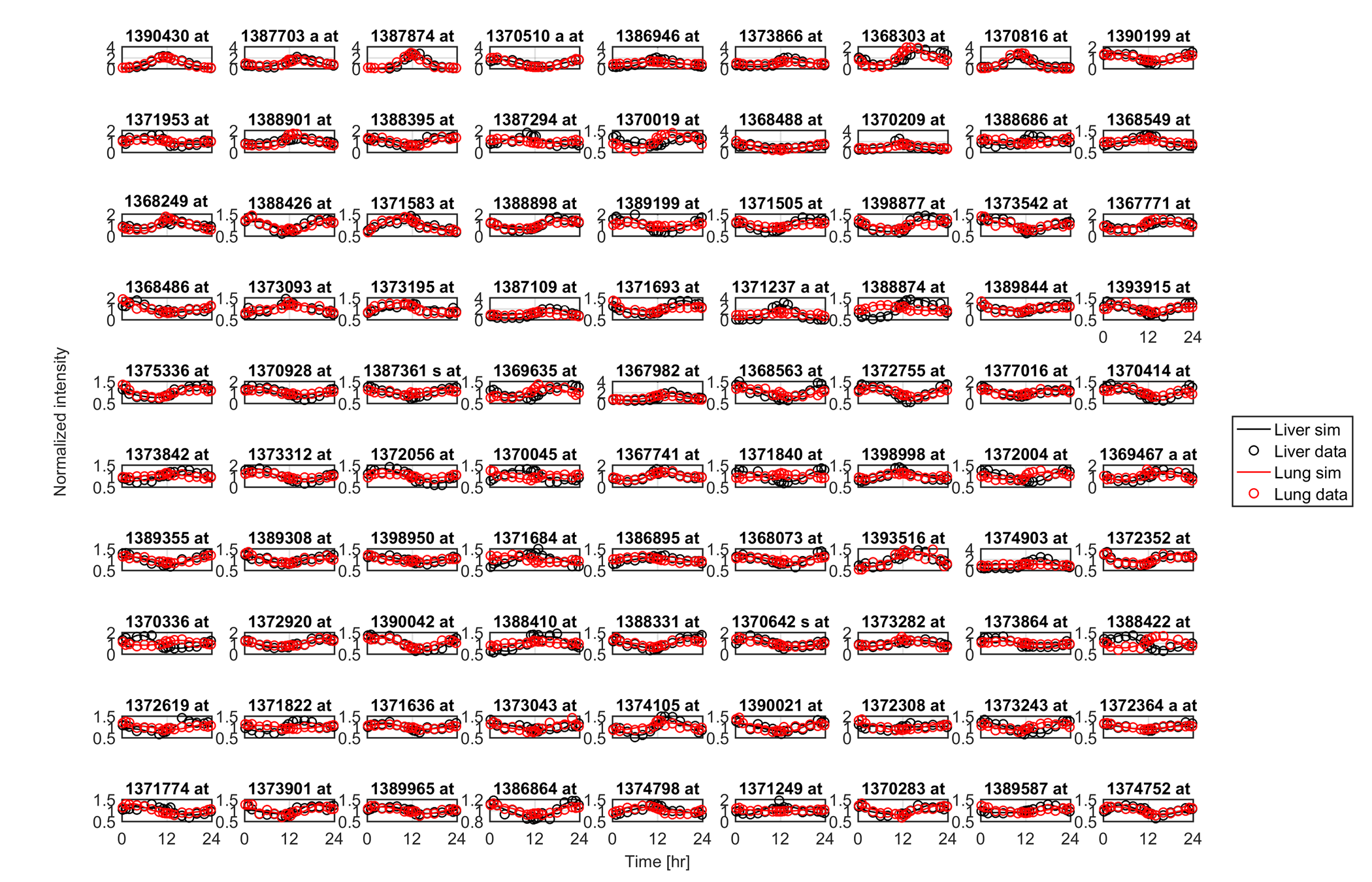

Supplement: S7 Fig — (TIF) [file pone.0197534.s007.tif]

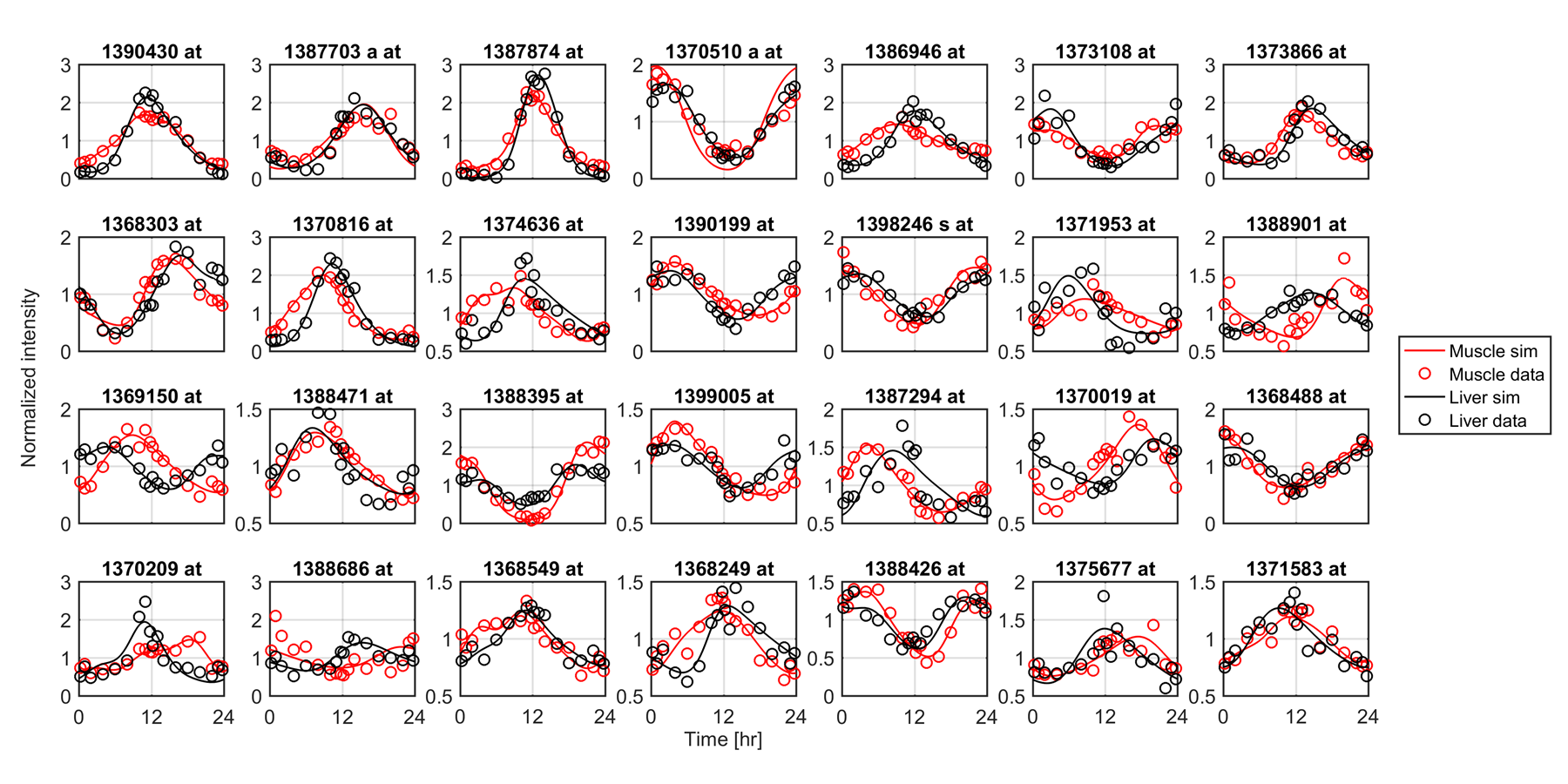

Supplement: S8 Fig — (TIF) [file pone.0197534.s008.tif]

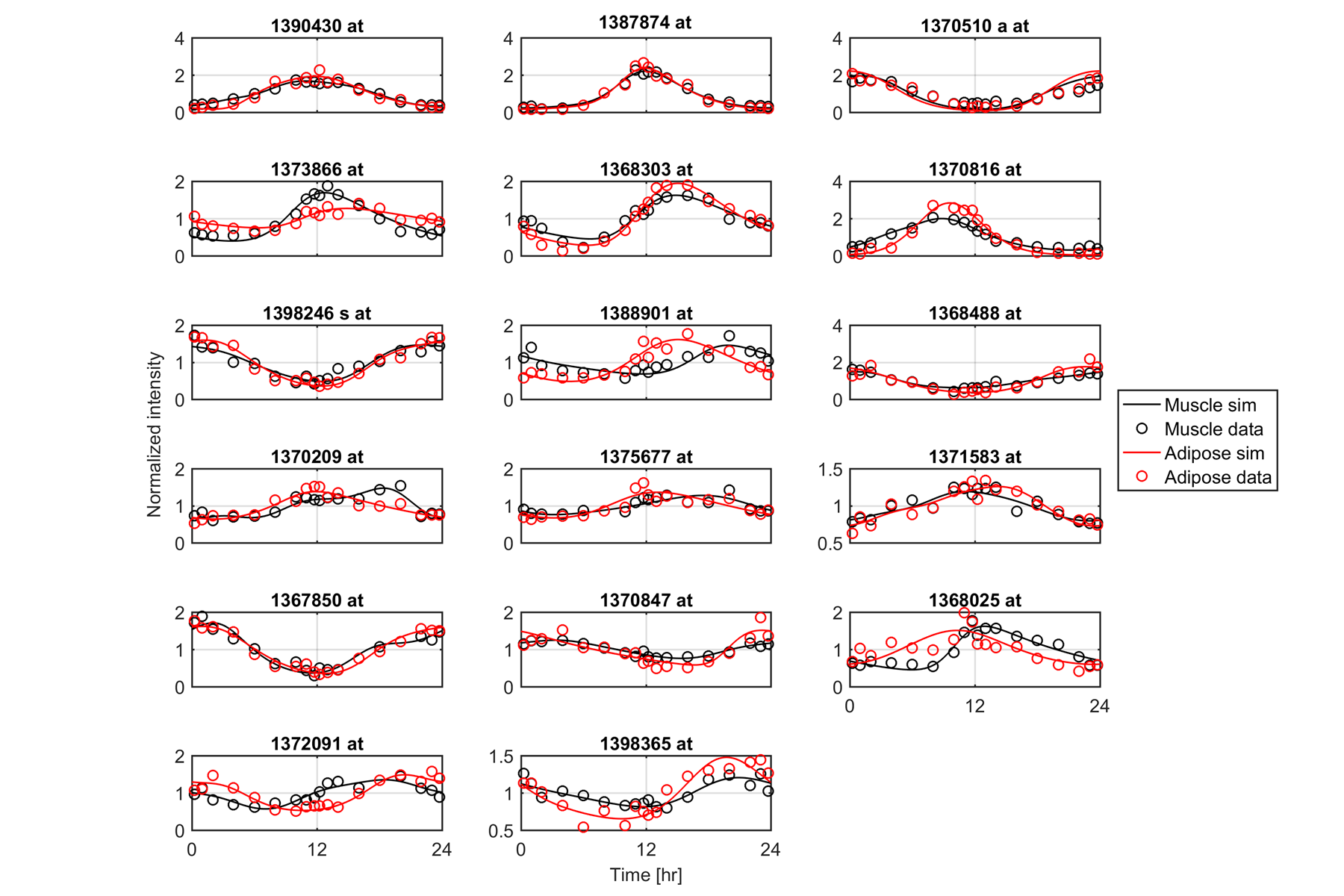

Supplement: S9 Fig — (TIF) [file pone.0197534.s009.tif]

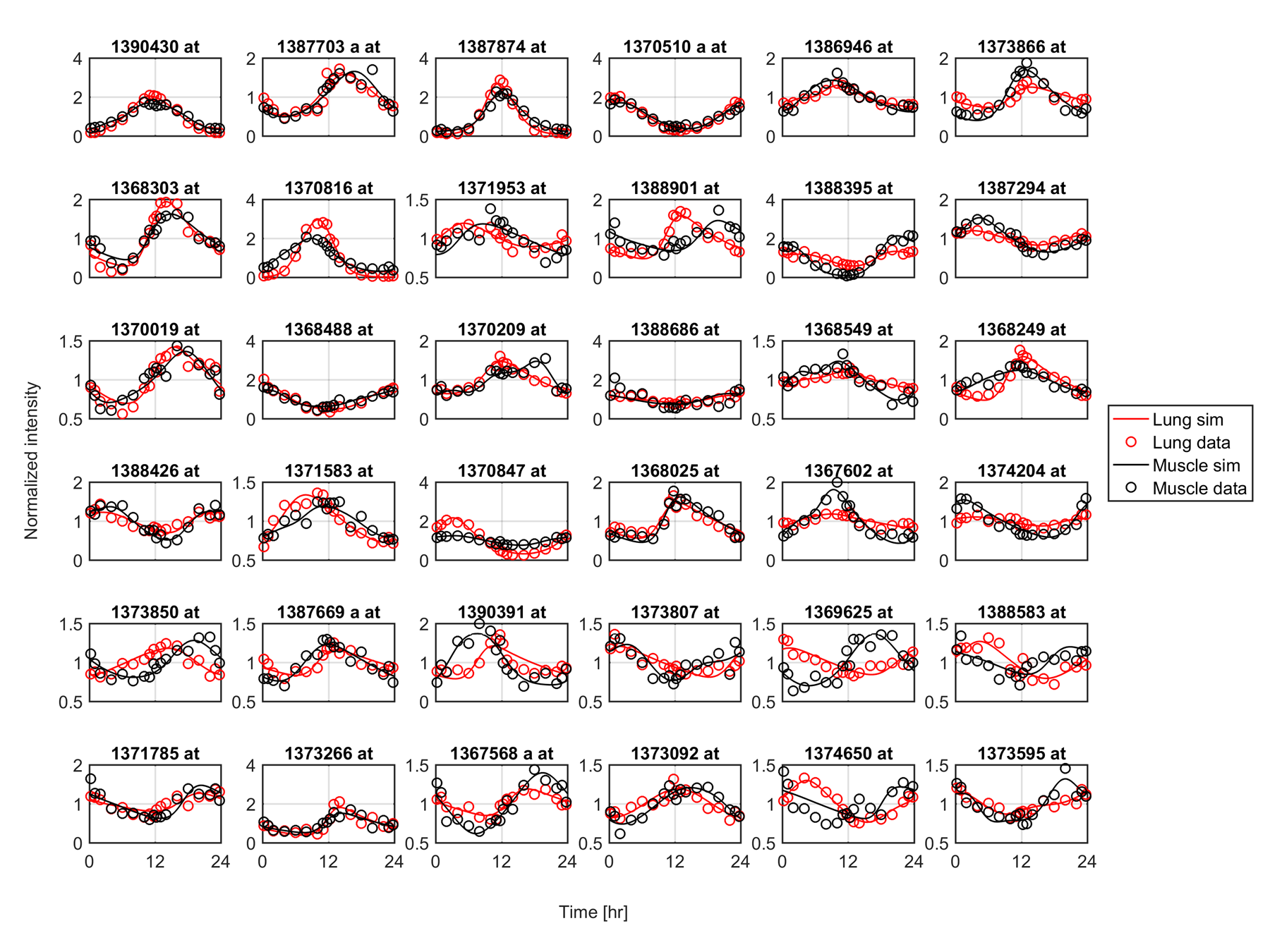

Supplement: S10 Fig — (TIF) [file pone.0197534.s010.tif]
