# Supplementary material for: Modeling circadian variability of core-clock and clock-controlled genes in four tissues of the rat
Source: PLoS One. 2018 Jun 12;13(6):e0197534. doi: 10.1371/journal.pone.0197534 (PMC5997360; doi:10.1371/journal.pone.0197534)
Supplement: S1 Appendix — (DOCX) [file pone.0197534.s011.docx]

# S1 Appendix: Transcription factor analysis and data fitting result tables

## Adipose-Lung

**Table A: Genes that are commonly oscillating in adipose and lung along with the optimized parameters used to fit their expression in the different tissues (Manuscript Equation 6).** Ebox/RRE/Dbox binding elements (n1, n2, n3) resulted from transcription factor binding site analysis at the promoter regions of the respective genes.

| ProbeIDs | Ebox | RRE | Dbox | Adipose | | | | | | | | Lung | | | | | | | | Δφ  (rads) |
| --- | --- | --- | --- | --- | --- | --- | --- | --- | --- | --- | --- | --- | --- | --- | --- | --- | --- | --- | --- | --- |
|  | **n1** | **n2** | **n3** | **b** | **ba** | **cr** | **gr** | **ar** | **f** | **fa** | **d** | **b** | **ba** | **cr** | **gr** | **ar** | **f** | **fa** | **d** |  |
| 1390430_at | 8 | 5 | 2 | 14.566 | 1.514 | 4.121 | 0.664 | 4.349 | 0.148 | 0.254 | 0.219 | 2.218 | 9.404 | 4.535 | 3.939 | 6.209 | 5.736 | 3.301 | 0.990 | 0.000 |
| 1387874_at | 4 | 2 | 2 | 2.441 | 1.720 | 0.639 | 3.573 | 1.217 | 16.191 | 16.738 | 0.951 | 1.169 | 3.034 | 0.529 | 14.063 | 9.026 | 9.809 | 14.403 | 0.998 | 0.000 |
| 1370510_a_at | 2 | 6 | 0 | 0.000 | 7.177 | 19.731 | 19.549 | 5.187 | NaN | NaN | 0.220 | 0.003 | 17.494 | 16.478 | 6.523 | 3.972 | NaN | NaN | 0.214 | 0.000 |
| 1373866_at | 3 | 2 | 1 | 14.314 | 14.011 | 1.268 | 0.329 | 20.000 | 20.000 | 7.240 | 0.044 | 17.404 | 2.976 | 0.174 | 0.687 | 19.926 | 20.000 | 16.150 | 0.040 | 0.000 |
| 1368303_at | 5 | 2 | 2 | 1.239 | 17.455 | 3.817 | 2.542 | 19.950 | 20.000 | 8.176 | 0.175 | 13.695 | 0.940 | 1.241 | 0.058 | 19.999 | 13.672 | 2.021 | 0.132 | 0.196 |
| 1370816_at | 7 | 2 | 1 | 1.379 | 12.569 | 2.641 | 19.996 | 8.755 | 14.918 | 0.840 | 0.773 | 1.868 | 0.807 | 2.353 | 4.165 | 6.524 | 1.763 | 19.970 | 1.000 | 0.000 |
| 1388901_at | 8 | 4 | 0 | 4.127 | 0.008 | 6.016 | 2.030 | 19.492 | NaN | NaN | 0.124 | 13.909 | 1.764 | 1.694 | 0.258 | 17.953 | NaN | NaN | 0.078 | 0.393 |
| 1368488_at | 1 | 2 | 2 | 6.886 | 19.998 | 2.741 | 1.774 | 1.187 | 2.103 | 9.663 | 0.373 | 19.562 | 1.945 | 2.434 | 19.413 | 1.221 | 14.008 | 2.895 | 0.277 | 0.000 |
| 1370209_at | 7 | 2 | 1 | 10.597 | 2.724 | 2.301 | 2.492 | 0.080 | 5.856 | 19.974 | 0.068 | 13.212 | 1.023 | 1.476 | 3.487 | 0.019 | 19.524 | 12.173 | 0.076 | 0.196 |
| 1371583_at | 1 | 5 | 1 | 19.351 | 9.247 | 0.702 | 4.248 | 19.043 | 6.369 | 14.940 | 1.000 | 18.172 | 2.274 | 0.741 | 19.141 | 18.586 | 0.367 | 19.998 | 0.116 | 0.589 |
| 1388898_at | 3 | 4 | 0 | 0.000 | 2.323 | 19.813 | 8.089 | 20.000 | NaN | NaN | 0.169 | 10.783 | 0.042 | 19.711 | 0.339 | 19.969 | NaN | NaN | 0.087 | 0.589 |
| 1389199_at | 3 | 5 | 2 | 0.000 | 1.587 | 4.027 | 9.730 | 6.558 | 7.252 | 5.152 | 0.067 | 11.385 | 1.655 | 5.673 | 2.790 | 3.495 | 3.098 | 17.544 | 0.087 | 0.982 |
| 1371505_at | 2 | 2 | 1 | 4.347 | 0.203 | 6.426 | 17.863 | 4.498 | 20.000 | 8.660 | 0.124 | 5.662 | 0.442 | 9.763 | 19.460 | 4.996 | 19.992 | 3.801 | 0.244 | 0.393 |
| 1398877_at | 1 | 1 | 0 | 0.002 | 0.940 | 9.493 | 1.362 | 5.325 | NaN | NaN | 0.154 | 0.000 | 1.369 | 10.999 | 4.322 | 19.221 | NaN | NaN | 0.173 | 0.393 |
| 1373542_at | 9 | 2 | 0 | 3.376 | 0.182 | 16.477 | 3.257 | 0.169 | NaN | NaN | 0.208 | 7.220 | 0.190 | 16.781 | 2.713 | 0.001 | NaN | NaN | 0.077 | 0.196 |
| 1367771_at | 1 | 4 | 0 | 0.001 | 3.967 | 2.281 | 0.197 | 20.000 | NaN | NaN | 0.448 | 12.822 | 0.000 | 0.702 | 0.027 | 20.000 | NaN | NaN | 0.386 | 0.589 |
| 1368486_at | 4 | 2 | 1 | 6.695 | 0.108 | 19.961 | 18.491 | 2.699 | 7.430 | 3.346 | 0.446 | 12.917 | 0.464 | 19.831 | 19.627 | 1.949 | 19.998 | 4.799 | 0.994 | 0.196 |
| 1373093_at | 7 | 6 | 0 | 15.151 | 1.480 | 0.676 | 0.564 | 19.937 | NaN | NaN | 0.062 | 8.875 | 0.425 | 15.805 | 6.173 | 18.832 | NaN | NaN | 0.044 | 0.589 |
| 1373195_at | 3 | 0 | 0 | 2.353 | 18.273 | 20.000 | 20.000 | NaN | NaN | NaN | 1.000 | 1.457 | 19.091 | 20.000 | 20.000 | NaN | NaN | NaN | 1.000 | 0.393 |
| 1370847_at | 7 | 5 | 0 | 8.650 | 0.000 | 19.989 | 0.558 | 0.154 | NaN | NaN | 0.061 | 15.448 | 0.181 | 19.283 | 19.401 | 2.109 | NaN | NaN | 0.211 | 0.393 |
| 1368025_at | 4 | 0 | 1 | 5.392 | 0.093 | 20.000 | 7.845 | NaN | 0.000 | 20.000 | 0.253 | 14.931 | 2.984 | 0.176 | 1.757 | NaN | 11.124 | 11.511 | 0.065 | 0.589 |
| 1378745_at | 4 | 3 | 3 | 10.395 | 16.240 | 1.562 | 1.533 | 2.656 | 2.968 | 7.309 | 0.921 | 1.434 | 0.490 | 1.417 | 4.025 | 11.530 | 2.070 | 20.000 | 0.510 | 0.000 |
| 1383439_at | 7 | 4 | 0 | 10.576 | 0.108 | 19.315 | 18.750 | 1.258 | NaN | NaN | 0.225 | 9.635 | 0.177 | 19.997 | 18.883 | 1.164 | NaN | NaN | 0.233 | 0.196 |
| 1390819_at | 11 | 7 | 1 | 2.107 | 3.708 | 4.988 | 5.029 | 16.190 | 7.157 | 17.136 | 0.096 | 2.192 | 0.084 | 3.113 | 9.742 | 18.669 | 12.088 | 19.990 | 0.112 | 0.000 |
| 1392640_at | 2 | 3 | 0 | 0.000 | 0.777 | 20.000 | 20.000 | 19.980 | NaN | NaN | 0.310 | 0.000 | 3.333 | 19.073 | 1.566 | 20.000 | NaN | NaN | 0.181 | 0.393 |
| 1398597_at | 3 | 2 | 0 | 9.629 | 5.466 | 13.331 | 3.164 | 0.714 | NaN | NaN | 0.213 | 3.043 | 0.226 | 12.963 | 4.901 | 1.541 | NaN | NaN | 0.154 | 0.393 |
| 1377635_at | 5 | 5 | 3 | 3.292 | 13.387 | 4.184 | 2.383 | 14.162 | 20.000 | 1.429 | 0.131 | 2.889 | 19.992 | 20.000 | 10.398 | 19.999 | 19.956 | 4.314 | 0.355 | 0.393 |
| 1393730_at | 5 | 9 | 0 | 2.915 | 0.000 | 17.822 | 2.997 | 6.344 | NaN | NaN | 0.141 | 5.692 | 0.108 | 16.994 | 7.925 | 7.574 | NaN | NaN | 0.225 | 0.393 |
| 1368304_at | 2 | 6 | 0 | 19.522 | 0.402 | 0.515 | 0.207 | 20.000 | NaN | NaN | 0.142 | 17.795 | 0.164 | 1.041 | 0.066 | 20.000 | NaN | NaN | 0.419 | 0.000 |
| 1378156_at | 1 | 2 | 2 | 19.999 | 5.873 | 0.807 | 2.533 | 3.815 | 4.220 | 12.320 | 0.972 | 19.899 | 2.599 | 0.277 | 20.000 | 19.673 | 5.804 | 20.000 | 0.136 | 0.000 |
| 1368247_at | 0 | 3 | 0 | 1.500 | 1.500 | 1.500 | 1.500 | 20.000 | NaN | 1.500 | 1.000 | 6.392 | 4.616 | 1.500 | 1.500 | 1.500 | 1.500 | 1.500 | 0.500 | 0.393 |
| 1368177_at | 2 | 3 | 1 | 3.643 | 0.280 | 18.619 | 14.642 | 12.501 | 19.902 | 19.999 | 0.208 | 9.242 | 0.696 | 8.877 | 13.547 | 6.999 | 19.997 | 7.473 | 0.153 | 0.982 |
| 1370266_at | 10 | 2 | 1 | 2.319 | 4.955 | 19.997 | 13.960 | 0.577 | 18.259 | 2.990 | 0.313 | 2.503 | 0.000 | 19.777 | 7.524 | 3.758 | 4.363 | 3.508 | 0.278 | 2.945 |
| 1371832_at | 2 | 4 | 0 | 0.003 | 2.122 | 15.825 | 12.682 | 17.077 | NaN | NaN | 0.152 | 0.745 | 2.010 | 11.751 | 3.183 | 7.276 | NaN | NaN | 0.163 | 0.000 |
| 1398635_at | 5 | 6 | 1 | 9.975 | 1.020 | 0.251 | 3.605 | 19.999 | 18.265 | 19.991 | 0.062 | 5.765 | 0.606 | 0.905 | 0.734 | 19.051 | 19.993 | 19.998 | 0.050 | 0.393 |
| 1373629_at | 3 | 4 | 0 | 15.257 | 0.834 | 1.747 | 0.233 | 19.054 | NaN | NaN | 0.061 | 19.396 | 0.438 | 1.192 | 0.083 | 20.000 | NaN | NaN | 0.022 | 0.393 |
| 1368200_at | 1 | 3 | 1 | 19.114 | 0.280 | 19.711 | 20.000 | 6.871 | 1.649 | 3.778 | 0.260 | 19.989 | 1.548 | 18.115 | 19.269 | 10.891 | 2.824 | 1.270 | 0.823 | 0.393 |
| 1398662_at | 4 | 4 | 2 | 17.538 | 0.331 | 20.000 | 20.000 | 7.291 | 20.000 | 5.046 | 0.284 | 3.597 | 0.264 | 4.322 | 19.988 | 6.042 | 20.000 | 3.704 | 0.358 | 1.767 |
| 1391187_at | 1 | 4 | 1 | 19.992 | 2.170 | 0.628 | 0.985 | 20.000 | 19.887 | 19.883 | 0.999 | 19.856 | 2.020 | 3.866 | 20.000 | 19.989 | 8.630 | 15.748 | 1.000 | 0.196 |
| 1368021_at | 3 | 5 | 0 | 19.943 | 1.115 | 0.657 | 0.192 | 20.000 | NaN | NaN | 0.350 | 6.423 | 0.000 | 2.920 | 1.388 | 10.004 | NaN | NaN | 0.207 | 0.589 |
| 1370570_at | 2 | 2 | 0 | 9.578 | 4.206 | 3.854 | 8.366 | 1.048 | NaN | NaN | 0.118 | 19.999 | 0.964 | 18.878 | 4.837 | 2.367 | NaN | NaN | 0.109 | 0.196 |
| 1379971_at | 5 | 4 | 1 | 6.097 | 20.000 | 16.739 | 19.889 | 9.610 | 20.000 | 1.554 | 0.152 | 8.580 | 20.000 | 3.590 | 4.365 | 2.506 | 19.999 | 4.697 | 0.086 | 0.393 |
| 1375908_at | 3 | 4 | 0 | 5.109 | 0.094 | 10.755 | 4.790 | 4.472 | NaN | 1.500 | 0.152 | 4.183 | 0.013 | 8.115 | 3.299 | 4.540 | NaN | NaN | 0.080 | 0.196 |
| 1372390_at | 2 | 1 | 2 | 2.140 | 20.000 | 0.136 | 5.888 | 1.004 | 12.888 | 1.488 | 1.000 | 0.305 | 6.914 | 19.973 | 1.710 | 7.628 | 0.070 | 19.984 | 0.379 | 1.178 |
| 1369526_at | 3 | 4 | 2 | 20.000 | 0.271 | 20.000 | 20.000 | 20.000 | 0.000 | 20.000 | 0.572 | 9.846 | 20.000 | 0.759 | 1.602 | 2.881 | 19.990 | 4.699 | 0.568 | 1.963 |
| 1391208_at | 1 | 4 | 1 | 16.110 | 8.757 | 0.389 | 0.607 | 19.606 | 5.884 | 19.998 | 0.339 | 3.978 | 19.999 | 0.119 | 1.483 | 19.999 | 12.637 | 18.497 | 0.312 | 0.589 |
| 1393917_at | 2 | 4 | 1 | 2.871 | 11.764 | 9.279 | 1.786 | 19.999 | 19.747 | 5.654 | 0.299 | 17.981 | 0.001 | 0.323 | 0.441 | 19.998 | 19.990 | 8.988 | 0.111 | 0.196 |
| 1398255_at | 1 | 4 | 1 | 16.660 | 13.695 | 0.055 | 8.824 | 19.999 | 11.856 | 6.594 | 0.347 | 0.951 | 17.087 | 0.227 | 6.673 | 10.966 | 19.814 | 6.205 | 0.119 | 2.553 |
| 1368882_at | 3 | 3 | 2 | 3.750 | 0.504 | 0.726 | 19.997 | 0.690 | 11.518 | 2.414 | 0.083 | 3.319 | 5.813 | 3.443 | 5.772 | 2.984 | 19.564 | 1.112 | 0.287 | 0.000 |
| 1385904_at | 6 | 4 | 1 | 8.997 | 20.000 | 12.597 | 8.900 | 4.733 | 16.052 | 1.174 | 0.119 | 9.453 | 19.998 | 19.999 | 19.995 | 5.627 | 19.997 | 4.823 | 0.268 | 0.785 |
| 1398522_at | 3 | 6 | 0 | 18.937 | 2.311 | 1.706 | 15.916 | 1.066 | NaN | NaN | 0.049 | 16.890 | 19.999 | 4.039 | 1.275 | 16.581 | NaN | NaN | 0.060 | 1.963 |

## Liver-Adipose

**Table B: Genes that are commonly oscillating in liver and adipose along with the optimized parameters used to fit their expression in the different tissues (Manuscript Equation 6).** Ebox/RRE/Dbox binding elements (n1, n2, n3) resulted from transcription factor binding site analysis at the promoter regions of the respective genes.

| ProbeIDs | Ebox | RRE | Dbox | Liver | | | | | | | | Adipose | | | | | | | | Δφ  (rads) |
| --- | --- | --- | --- | --- | --- | --- | --- | --- | --- | --- | --- | --- | --- | --- | --- | --- | --- | --- | --- | --- |
|  | **n1** | **n2** | **n3** | **b** | **ba** | **cr** | **gr** | **ar** | **f** | **fa** | **d** | **b** | **ba** | **cr** | **gr** | **ar** | **f** | **fa** | **d** |  |
| 1390430_at | 8 | 5 | 2 | 5.348 | 10.000 | 2.500 | 2.163 | 4.538 | 9.065 | 1.588 | 0.233 | 14.566 | 1.514 | 4.121 | 0.664 | 4.349 | 0.148 | 0.254 | 0.219 | 0.196 |
| 1387874_at | 4 | 2 | 2 | 5.846 | 0.324 | 0.598 | 0.146 | 3.899 | 11.999 | 2.883 | 0.898 | 2.441 | 1.720 | 0.639 | 3.573 | 1.217 | 16.191 | 16.738 | 0.951 | 0.196 |
| 1370510_a_at | 2 | 6 | 0 | 5.638 | 11.937 | 15.660 | 2.923 | 6.139 | 1.500 | NaN | 0.254 | 0.000 | 7.177 | 19.731 | 19.549 | 5.187 | NaN | NaN | 0.220 | 0.196 |
| 1373866_at | 3 | 2 | 1 | 19.956 | 1.804 | 1.249 | 0.059 | 19.989 | 19.981 | 19.999 | 0.132 | 14.314 | 14.011 | 1.268 | 0.329 | 20.000 | 20.000 | 7.240 | 0.044 | 0.000 |
| 1368303_at | 5 | 2 | 2 | 19.192 | 0.302 | 20.000 | 20.000 | 1.685 | 19.914 | 11.958 | 0.772 | 1.239 | 17.455 | 3.817 | 2.542 | 19.950 | 20.000 | 8.176 | 0.175 | 0.589 |
| 1370816_at | 7 | 2 | 1 | 3.744 | 19.995 | 1.816 | 19.649 | 2.359 | 19.999 | 3.742 | 0.297 | 1.379 | 12.569 | 2.641 | 19.996 | 8.755 | 14.918 | 0.840 | 0.773 | 0.196 |
| 1398246_s_at | 8 | 7 | 1 | 8.826 | 0.207 | 20.000 | 4.329 | 3.934 | 4.877 | 6.876 | 0.121 | 15.840 | 0.080 | 19.990 | 19.994 | 6.146 | 19.737 | 9.637 | 0.324 | 0.196 |
| 1388901_at | 8 | 4 | 0 | 6.550 | 0.291 | 18.632 | 4.549 | 12.292 | NaN | NaN | 0.078 | 4.127 | 0.008 | 6.016 | 2.030 | 19.492 | NaN | NaN | 0.124 | 0.589 |
| 1368488_at | 1 | 2 | 2 | 19.824 | 0.062 | 19.985 | 15.050 | 5.600 | 1.118 | 7.702 | 0.289 | 6.886 | 19.998 | 2.741 | 1.774 | 1.187 | 2.103 | 9.663 | 0.373 | 0.000 |
| 1370209_at | 7 | 2 | 1 | 19.935 | 13.395 | 10.796 | 1.458 | 0.043 | 20.000 | 9.262 | 1.000 | 10.597 | 2.724 | 2.301 | 2.492 | 0.080 | 5.856 | 19.974 | 0.068 | 0.393 |
| 1375677_at | 2 | 2 | 0 | 17.807 | 3.767 | 0.250 | 3.285 | 19.260 | NaN | NaN | 0.073 | 15.990 | 2.530 | 0.471 | 0.615 | 18.438 | NaN | NaN | 0.076 | 0.196 |
| 1371583_at | 1 | 5 | 1 | 20.000 | 10.088 | 0.249 | 1.133 | 20.000 | 0.000 | 19.998 | 0.094 | 19.351 | 9.247 | 0.702 | 4.248 | 19.043 | 6.369 | 14.940 | 1.000 | 0.196 |
| 1373718_at | 2 | 5 | 2 | 20.000 | 0.000 | 20.000 | 4.572 | 6.229 | 0.000 | 20.000 | 0.274 | 19.599 | 4.404 | 0.421 | 19.770 | 3.953 | 7.384 | 8.637 | 0.464 | 0.393 |
| 1388898_at | 3 | 4 | 0 | 11.403 | 0.094 | 19.887 | 0.093 | 16.125 | NaN | NaN | 0.126 | 0.000 | 2.323 | 19.813 | 8.089 | 20.000 | NaN | NaN | 0.169 | 0.785 |
| 1389199_at | 3 | 5 | 2 | 5.423 | 19.985 | 17.823 | 10.133 | 5.331 | 20.000 | 0.000 | 0.253 | 0.000 | 1.587 | 4.027 | 9.730 | 6.558 | 7.252 | 5.152 | 0.067 | 0.785 |
| 1371505_at | 2 | 2 | 1 | 10.442 | 0.451 | 10.113 | 9.721 | 3.948 | 18.159 | 13.269 | 0.316 | 4.347 | 0.203 | 6.426 | 17.863 | 4.498 | 20.000 | 8.660 | 0.124 | 0.393 |
| 1398877_at | 1 | 1 | 0 | 0.000 | 1.411 | 19.754 | 2.726 | 13.689 | NaN | NaN | 1.000 | 0.002 | 0.940 | 9.493 | 1.362 | 5.325 | NaN | NaN | 0.154 | 0.785 |
| 1373542_at | 9 | 2 | 0 | 3.123 | 0.005 | 17.672 | 12.373 | 1.171 | NaN | NaN | 0.226 | 3.376 | 0.182 | 16.477 | 3.257 | 0.169 | NaN | NaN | 0.208 | 0.589 |
| 1367771_at | 1 | 4 | 0 | 0.712 | 4.497 | 2.319 | 0.193 | 19.989 | NaN | NaN | 0.292 | 0.001 | 3.967 | 2.281 | 0.197 | 20.000 | NaN | NaN | 0.448 | 0.196 |
| 1368486_at | 4 | 2 | 1 | 20.000 | 13.195 | 19.998 | 2.150 | 0.684 | 2.537 | 8.401 | 0.978 | 6.695 | 0.108 | 19.961 | 18.491 | 2.699 | 7.430 | 3.346 | 0.446 | 0.785 |
| 1373093_at | 7 | 6 | 0 | 14.513 | 3.577 | 0.540 | 5.208 | 19.993 | NaN | NaN | 0.032 | 15.151 | 1.480 | 0.676 | 0.564 | 19.937 | NaN | NaN | 0.062 | 0.393 |
| 1373195_at | 3 | 0 | 0 | 2.412 | 8.986 | 19.939 | 19.999 | NaN | NaN | NaN | 0.052 | 2.353 | 18.273 | 20.000 | 20.000 | NaN | NaN | NaN | 1.000 | 0.000 |

## Liver-Lung

**Table C: Genes that are commonly oscillating in liver and lung along with the optimized parameters used to fit their expression in the different tissues (Manuscript Equation 6** Ebox/RRE/Dbox binding elements (n1, n2, n3) resulted from transcription factor binding site analysis at the promoter regions of the respective genes.

| ProbeIDs | Ebox | RRE | Dbox | Liver | | | | | | | | Lung | | | | | | | | Δφ  (rads) |
| --- | --- | --- | --- | --- | --- | --- | --- | --- | --- | --- | --- | --- | --- | --- | --- | --- | --- | --- | --- | --- |
|  | **n1** | **n2** | **n3** | **b** | **ba** | **cr** | **gr** | **ar** | **f** | **fa** | **d** | **b** | **ba** | **cr** | **gr** | **ar** | **f** | **fa** | **d** |  |
| 1390430_at | 8 | 5 | 2 | 5.348 | 10.000 | 2.500 | 2.163 | 4.538 | 9.065 | 1.588 | 0.233 | 2.218 | 9.404 | 4.535 | 3.939 | 6.209 | 5.736 | 3.301 | 0.990 | 0.196 |
| 1387703_a_at | 3 | 4 | 0 | 19.993 | 0.108 | 1.424 | 0.069 | 20.000 | NaN | NaN | 0.245 | 19.962 | 0.541 | 1.456 | 0.117 | 20.000 | NaN | NaN | 0.120 | 0.000 |
| 1387874_at | 4 | 2 | 2 | 5.846 | 0.324 | 0.598 | 0.146 | 3.899 | 11.999 | 2.883 | 0.898 | 1.169 | 3.034 | 0.529 | 14.063 | 9.026 | 9.809 | 14.403 | 0.998 | 0.196 |
| 1370510_a_at | 2 | 6 | 0 | 5.638 | 11.937 | 15.660 | 2.923 | 6.139 | NaN | NaN | 0.254 | 0.003 | 17.494 | 16.478 | 6.523 | 3.972 | NaN | NaN | 0.214 | 0.196 |
| 1386946_at | 4 | 1 | 1 | 5.227 | 18.199 | 1.120 | 2.267 | 1.222 | 20.000 | 1.190 | 0.173 | 2.461 | 19.750 | 4.523 | 6.035 | 0.400 | 12.740 | 1.903 | 1.000 | 0.589 |
| 1373866_at | 3 | 2 | 1 | 19.956 | 1.804 | 1.249 | 0.059 | 19.989 | 19.981 | 19.999 | 0.132 | 17.404 | 2.976 | 0.174 | 0.687 | 19.926 | 20.000 | 16.150 | 0.040 | 0.000 |
| 1368303_at | 5 | 2 | 2 | 19.192 | 0.302 | 20.000 | 20.000 | 1.685 | 19.914 | 11.958 | 0.772 | 13.695 | 0.940 | 1.241 | 0.058 | 19.999 | 13.672 | 2.021 | 0.132 | 0.785 |
| 1370816_at | 7 | 2 | 1 | 3.744 | 19.995 | 1.816 | 19.649 | 2.359 | 19.999 | 3.742 | 0.297 | 1.868 | 0.807 | 2.353 | 4.165 | 6.524 | 1.763 | 19.970 | 1.000 | 0.196 |
| 1390199_at | 5 | 4 | 0 | 3.969 | 0.000 | 13.633 | 7.003 | 3.402 | NaN | NaN | 0.163 | 1.938 | 0.042 | 11.911 | 3.565 | 1.805 | NaN | NaN | 0.073 | 0.196 |
| 1371953_at | 4 | 5 | 0 | 7.967 | 19.981 | 20.000 | 14.112 | 8.841 | NaN | NaN | 0.171 | 2.637 | 1.092 | 7.817 | 3.756 | 3.259 | NaN | NaN | 0.044 | 0.196 |
| 1388901_at | 8 | 4 | 0 | 6.550 | 0.291 | 18.632 | 4.549 | 12.292 | NaN | NaN | 0.078 | 13.909 | 1.764 | 1.694 | 0.258 | 17.953 | NaN | NaN | 0.078 | 0.196 |
| 1388395_at | 4 | 6 | 0 | 4.980 | 0.000 | 20.000 | 5.415 | 13.102 | NaN | NaN | 1.000 | 3.456 | 0.213 | 10.784 | 1.798 | 6.152 | NaN | NaN | 0.192 | 0.393 |
| 1387294_at | 12 | 5 | 1 | 4.198 | 19.578 | 19.988 | 19.991 | 5.929 | 19.990 | 3.551 | 0.172 | 1.165 | 0.125 | 19.996 | 20.000 | 3.201 | 20.000 | 4.312 | 0.058 | 1.571 |
| 1370019_at | 6 | 3 | 0 | 2.667 | 0.000 | 16.487 | 1.318 | 3.867 | NaN | NaN | 0.043 | 9.367 | 1.627 | 8.477 | 0.236 | 13.722 | NaN | NaN | 0.057 | 1.374 |
| 1368488_at | 1 | 2 | 2 | 19.824 | 0.062 | 19.985 | 15.050 | 5.600 | 1.118 | 7.702 | 0.289 | 19.562 | 1.945 | 2.434 | 19.413 | 1.221 | 14.008 | 2.895 | 0.277 | 0.000 |
| 1370209_at | 7 | 2 | 1 | 19.935 | 13.395 | 10.796 | 1.458 | 0.043 | 20.000 | 9.262 | 1.000 | 13.212 | 1.023 | 1.476 | 3.487 | 0.019 | 19.524 | 12.173 | 0.076 | 0.589 |
| 1388686_at | 2 | 0 | 1 | 7.968 | 0.409 | 20.000 | 18.611 | NaN | 19.570 | 9.266 | 0.176 | 0.000 | 0.161 | 19.910 | 18.029 | NaN | 15.558 | 0.000 | 0.050 | 1.963 |
| 1368549_at | 2 | 2 | 1 | 3.400 | 8.114 | 0.355 | 19.429 | 2.571 | 19.888 | 7.127 | 0.352 | 4.310 | 4.534 | 0.799 | 14.010 | 2.067 | 19.379 | 7.168 | 0.140 | 0.000 |
| 1368249_at | 6 | 2 | 0 | 8.982 | 3.668 | 1.511 | 0.778 | 9.909 | NaN | NaN | 0.042 | 13.658 | 1.985 | 0.684 | 0.581 | 12.412 | NaN | NaN | 0.080 | 0.393 |
| 1388426_at | 3 | 10 | 0 | 12.570 | 0.157 | 20.000 | 2.217 | 20.000 | NaN | NaN | 0.239 | 11.671 | 0.536 | 16.378 | 0.429 | 7.043 | NaN | NaN | 0.069 | 0.196 |
| 1371583_at | 1 | 5 | 1 | 20.000 | 10.088 | 0.249 | 1.133 | 20.000 | 0.000 | 19.998 | 0.094 | 18.172 | 2.274 | 0.741 | 19.141 | 18.586 | 0.367 | 19.998 | 0.116 | 0.393 |
| 1388898_at | 3 | 4 | 0 | 11.403 | 0.094 | 19.887 | 0.093 | 16.125 | NaN | NaN | 0.126 | 10.783 | 0.042 | 19.711 | 0.339 | 19.969 | NaN | NaN | 0.087 | 0.196 |
| 1389199_at | 3 | 5 | 2 | 5.423 | 19.985 | 17.823 | 10.133 | 5.331 | 20.000 | 0.000 | 0.253 | 11.385 | 1.655 | 5.673 | 2.790 | 3.495 | 3.098 | 17.544 | 0.087 | 0.196 |
| 1371505_at | 2 | 2 | 1 | 10.442 | 0.451 | 10.113 | 9.721 | 3.948 | 18.159 | 13.269 | 0.316 | 5.662 | 0.442 | 9.763 | 19.460 | 4.996 | 19.992 | 3.801 | 0.244 | 0.785 |
| 1398877_at | 1 | 1 | 0 | 0.000 | 1.411 | 19.754 | 2.726 | 13.689 | NaN | NaN | 1.000 | 0.000 | 1.369 | 10.999 | 4.322 | 19.221 | NaN | NaN | 0.173 | 0.393 |
| 1373542_at | 9 | 2 | 0 | 3.123 | 0.005 | 17.672 | 12.373 | 1.171 | NaN | NaN | 0.226 | 7.220 | 0.190 | 16.781 | 2.713 | 0.001 | NaN | NaN | 0.077 | 0.393 |
| 1367771_at | 1 | 4 | 0 | 0.712 | 4.497 | 2.319 | 0.193 | 19.989 | NaN | NaN | 0.292 | 12.822 | 0.000 | 0.702 | 0.027 | 20.000 | NaN | NaN | 0.386 | 0.393 |
| 1368486_at | 4 | 2 | 1 | 20.000 | 13.195 | 19.998 | 2.150 | 0.684 | 2.537 | 8.401 | 0.978 | 12.917 | 0.464 | 19.831 | 19.627 | 1.949 | 19.998 | 4.799 | 0.994 | 0.589 |
| 1373093_at | 7 | 6 | 0 | 14.513 | 3.577 | 0.540 | 5.208 | 19.993 | NaN | NaN | 0.032 | 8.875 | 0.425 | 15.805 | 6.173 | 18.832 | NaN | NaN | 0.044 | 0.196 |
| 1373195_at | 3 | 0 | 0 | 2.412 | 8.986 | 19.939 | 19.999 | NaN | NaN | NaN | 0.052 | 1.457 | 19.091 | 20.000 | 20.000 | NaN | NaN | NaN | 1.000 | 0.393 |
| 1387109_at | 6 | 4 | 1 | 1.611 | 1.104 | 19.997 | 1.582 | 19.997 | 19.877 | 7.235 | 0.253 | 19.970 | 0.441 | 20.000 | 20.000 | 18.222 | 20.000 | 6.963 | 0.103 | 0.785 |
| 1371693_at | 6 | 5 | 1 | 2.220 | 0.095 | 19.628 | 3.276 | 17.381 | 19.481 | 7.904 | 0.086 | 2.190 | 0.001 | 19.962 | 17.855 | 11.582 | 19.954 | 8.266 | 0.082 | 0.785 |
| 1371237_a_at | 8 | 5 | 2 | 20.000 | 0.502 | 16.950 | 2.562 | 9.234 | 20.000 | 2.563 | 1.000 | 3.350 | 0.104 | 3.666 | 11.845 | 12.522 | 1.601 | 0.018 | 0.047 | 0.000 |
| 1388874_at | 4 | 3 | 0 | 6.449 | 0.000 | 3.268 | 0.976 | 17.182 | NaN | NaN | 0.102 | 2.686 | 1.609 | 7.653 | 4.695 | 2.361 | NaN | NaN | 0.057 | 2.160 |
| 1389844_at | 4 | 5 | 0 | 6.964 | 0.000 | 19.069 | 0.833 | 8.220 | NaN | NaN | 0.075 | 9.189 | 0.000 | 19.967 | 0.685 | 6.211 | NaN | NaN | 0.059 | 0.196 |
| 1393915_at | 4 | 6 | 0 | 1.693 | 0.045 | 12.759 | 1.789 | 3.807 | NaN | NaN | 0.061 | 3.654 | 0.508 | 9.503 | 1.847 | 5.870 | NaN | NaN | 0.113 | 0.393 |
| 1375336_at | 4 | 4 | 0 | 8.298 | 0.072 | 16.374 | 0.502 | 17.397 | NaN | NaN | 0.065 | 8.538 | 0.002 | 18.334 | 0.492 | 6.614 | NaN | NaN | 0.038 | 0.196 |
| 1370928_at | 4 | 5 | 2 | 0.588 | 0.386 | 2.736 | 12.647 | 1.682 | 13.351 | 0.001 | 0.109 | 1.980 | 0.958 | 8.584 | 2.127 | 3.751 | 2.840 | 0.013 | 0.081 | 0.196 |
| 1387361_s_at | 3 | 9 | 1 | 2.589 | 0.317 | 9.274 | 7.910 | 15.064 | 18.745 | 9.621 | 0.112 | 1.633 | 0.823 | 6.290 | 6.859 | 20.000 | 12.235 | 10.425 | 0.055 | 0.589 |
| 1369635_at | 5 | 3 | 0 | 4.615 | 0.000 | 17.169 | 0.884 | 8.440 | NaN | NaN | 0.061 | 4.528 | 3.466 | 5.878 | 0.844 | 10.229 | NaN | NaN | 0.054 | 1.571 |
| 1367982_at | 5 | 2 | 1 | 19.925 | 1.020 | 6.189 | 0.048 | 15.991 | 5.174 | 20.000 | 0.122 | 18.768 | 1.216 | 1.135 | 1.207 | 0.073 | 19.667 | 14.520 | 0.106 | 0.785 |
| 1368563_at | 1 | 2 | 0 | 0.000 | 0.212 | 11.780 | 4.947 | 6.346 | NaN | NaN | 0.122 | 0.777 | 1.488 | 1.609 | 7.679 | 0.925 | NaN | NaN | 0.049 | 0.982 |
| 1372755_at | 2 | 7 | 1 | 2.706 | 8.247 | 4.894 | 1.021 | 6.103 | 6.493 | 0.001 | 0.111 | 2.756 | 10.078 | 3.577 | 1.655 | 6.383 | 6.986 | 0.001 | 0.059 | 0.393 |
| 1377016_at | 5 | 6 | 1 | 20.000 | 0.137 | 20.000 | 20.000 | 10.571 | 20.000 | 4.944 | 1.000 | 20.000 | 0.124 | 20.000 | 20.000 | 13.783 | 20.000 | 4.215 | 1.000 | 0.000 |
| 1370414_at | 4 | 5 | 3 | 1.461 | 10.788 | 8.456 | 2.186 | 2.061 | 3.065 | 0.000 | 0.074 | 3.164 | 7.857 | 10.675 | 3.010 | 5.819 | 16.029 | 0.026 | 0.126 | 0.393 |
| 1373842_at | 1 | 3 | 0 | 1.728 | 1.062 | 2.591 | 0.128 | 19.904 | NaN | NaN | 0.204 | 5.693 | 3.045 | 0.147 | 3.043 | 13.317 | NaN | NaN | 0.048 | 1.374 |
| 1373312_at | 2 | 2 | 0 | 5.494 | 12.283 | 4.996 | 9.643 | 0.579 | NaN | NaN | 0.162 | 4.540 | 0.494 | 8.218 | 5.620 | 1.343 | NaN | NaN | 0.100 | 0.000 |
| 1372056_at | 4 | 3 | 0 | 12.835 | 0.034 | 1.103 | 9.905 | 0.351 | NaN | NaN | 0.068 | 1.877 | 1.236 | 8.550 | 3.566 | 1.926 | NaN | NaN | 0.048 | 0.196 |
| 1370045_at | 6 | 3 | 1 | 11.096 | 19.989 | 5.113 | 3.930 | 0.834 | 20.000 | 5.396 | 0.135 | 19.999 | 0.160 | 20.000 | 20.000 | 4.598 | 20.000 | 7.239 | 0.274 | 2.553 |
| 1367741_at | 5 | 3 | 1 | 8.576 | 6.343 | 0.950 | 0.811 | 12.173 | 16.241 | 1.580 | 0.068 | 3.748 | 1.233 | 1.213 | 1.858 | 10.481 | 5.524 | 19.999 | 0.063 | 0.000 |
| 1371840_at | 3 | 1 | 1 | 0.227 | 0.160 | 19.958 | 19.994 | 1.696 | 19.999 | 0.000 | 0.095 | 3.176 | 11.447 | 4.016 | 2.006 | 0.201 | 19.274 | 0.752 | 0.482 | 3.142 |
| 1398998_at | 4 | 6 | 1 | 20.000 | 5.048 | 0.198 | 19.999 | 7.581 | 20.000 | 3.298 | 0.044 | 4.908 | 2.339 | 1.053 | 5.927 | 6.244 | 2.456 | 0.966 | 0.020 | 0.000 |
| 1372004_at | 5 | 6 | 1 | 1.153 | 1.976 | 12.427 | 4.433 | 5.039 | 12.952 | 9.288 | 0.066 | 1.181 | 0.652 | 4.062 | 2.946 | 19.991 | 19.994 | 18.501 | 0.038 | 1.571 |
| 1369467_a_at | 1 | 6 | 3 | 19.994 | 2.967 | 0.004 | 19.895 | 19.987 | 6.985 | 5.147 | 0.223 | 20.000 | 16.077 | 0.006 | 2.389 | 19.998 | 14.300 | 1.346 | 0.482 | 1.178 |
| 1389355_at | 3 | 2 | 0 | 0.002 | 2.835 | 17.849 | 13.651 | 8.045 | NaN | NaN | 0.112 | 0.162 | 2.775 | 15.864 | 7.017 | 3.258 | NaN | NaN | 0.052 | 0.393 |
| 1389308_at | 4 | 3 | 1 | 2.847 | 0.692 | 10.096 | 4.570 | 2.727 | 17.839 | 19.987 | 0.097 | 6.948 | 0.204 | 20.000 | 20.000 | 7.189 | 20.000 | 2.713 | 0.950 | 0.000 |
| 1398950_at | 1 | 4 | 2 | 0.000 | 8.822 | 0.588 | 2.986 | 5.355 | 1.425 | 1.040 | 0.063 | 20.000 | 2.939 | 0.002 | 19.291 | 7.710 | 17.334 | 2.064 | 0.310 | 0.982 |
| 1371684_at | 2 | 4 | 1 | 19.220 | 4.974 | 1.134 | 19.666 | 16.022 | 19.851 | 2.476 | 0.079 | 2.417 | 11.947 | 3.154 | 20.000 | 5.531 | 14.285 | 1.114 | 0.075 | 1.963 |
| 1386895_at | 2 | 7 | 4 | 13.341 | 3.219 | 0.894 | 13.181 | 12.653 | 2.005 | 6.160 | 0.119 | 7.299 | 4.014 | 0.234 | 11.986 | 7.073 | 2.503 | 1.615 | 0.067 | 1.374 |
| 1368073_at | 1 | 5 | 0 | 0.000 | 0.288 | 11.430 | 1.133 | 8.771 | NaN | NaN | 0.065 | 1.773 | 3.980 | 5.532 | 1.454 | 9.248 | NaN | NaN | 0.051 | 0.000 |
| 1393516_at | 2 | 3 | 0 | 10.928 | 0.952 | 2.360 | 0.218 | 15.842 | NaN | NaN | 0.110 | 10.419 | 0.000 | 1.896 | 0.825 | 15.705 | NaN | NaN | 0.194 | 0.589 |
| 1374903_at | 5 | 5 | 2 | 9.281 | 1.326 | 19.998 | 2.163 | 6.760 | 19.982 | 12.053 | 0.517 | 12.451 | 1.328 | 5.655 | 6.775 | 4.241 | 6.049 | 20.000 | 0.076 | 0.982 |
| 1372352_at | 7 | 3 | 0 | 10.714 | 0.370 | 16.820 | 0.250 | 2.102 | NaN | NaN | 0.031 | 5.463 | 0.032 | 18.567 | 0.917 | 5.387 | NaN | NaN | 0.033 | 0.196 |
| 1370336_at | 5 | 7 | 0 | 4.779 | 15.073 | 19.980 | 12.129 | 8.849 | NaN | NaN | 0.257 | 15.874 | 0.990 | 2.790 | 0.195 | 19.621 | NaN | NaN | 0.037 | 2.945 |
| 1372920_at | 5 | 1 | 1 | 0.644 | 0.244 | 10.220 | 18.007 | 0.458 | 19.878 | 2.966 | 0.613 | 0.649 | 0.254 | 11.899 | 6.650 | 0.570 | 19.739 | 0.955 | 0.288 | 0.000 |
| 1390042_at | 6 | 3 | 1 | 0.975 | 0.688 | 17.677 | 9.613 | 0.942 | 3.276 | 0.006 | 0.072 | 1.737 | 0.993 | 17.657 | 3.422 | 1.770 | 6.314 | 0.007 | 0.123 | 0.393 |
| 1388410_at | 4 | 1 | 3 | 10.075 | 12.578 | 19.467 | 1.035 | 0.028 | 1.994 | 1.002 | 1.000 | 2.725 | 7.174 | 7.961 | 3.599 | 0.412 | 1.356 | 1.550 | 0.105 | 0.589 |
| 1388331_at | 7 | 5 | 0 | 5.744 | 0.000 | 19.784 | 1.551 | 6.409 | NaN | NaN | 0.063 | 7.279 | 0.015 | 19.759 | 1.273 | 6.284 | NaN | NaN | 0.027 | 0.196 |
| 1370642_s_at | 5 | 4 | 0 | 3.706 | 2.902 | 18.205 | 3.024 | 2.297 | NaN | NaN | 0.192 | 2.620 | 0.305 | 12.876 | 5.541 | 0.352 | NaN | NaN | 0.049 | 0.000 |
| 1373282_at | 2 | 4 | 0 | 7.096 | 0.003 | 1.059 | 0.607 | 14.850 | NaN | NaN | 0.137 | 19.724 | 2.563 | 0.352 | 0.831 | 20.000 | NaN | NaN | 0.088 | 0.785 |
| 1373864_at | 3 | 3 | 0 | 3.119 | 0.676 | 6.702 | 5.494 | 1.826 | NaN | NaN | 0.171 | 2.405 | 1.137 | 6.571 | 4.321 | 2.342 | NaN | NaN | 0.059 | 0.393 |
| 1388422_at | 1 | 6 | 1 | 5.462 | 19.798 | 19.987 | 1.855 | 19.999 | 0.038 | 12.776 | 0.159 | 20.000 | 1.416 | 0.003 | 19.931 | 20.000 | 20.000 | 14.013 | 0.264 | 2.945 |
| 1372619_at | 2 | 3 | 0 | 0.000 | 3.860 | 10.851 | 6.610 | 20.000 | NaN | NaN | 1.000 | 0.000 | 1.283 | 14.594 | 7.867 | 14.231 | NaN | NaN | 0.045 | 0.982 |
| 1371822_at | 3 | 3 | 0 | 15.133 | 0.653 | 1.969 | 0.094 | 18.395 | NaN | NaN | 0.046 | 1.582 | 0.165 | 8.048 | 1.385 | 1.670 | NaN | NaN | 0.023 | 2.160 |
| 1371636_at | 2 | 6 | 0 | 5.560 | 0.270 | 8.424 | 2.146 | 10.188 | NaN | NaN | 0.087 | 2.314 | 4.121 | 4.550 | 1.935 | 6.496 | NaN | NaN | 0.039 | 0.393 |
| 1373043_at | 5 | 5 | 0 | 3.548 | 0.002 | 16.203 | 0.852 | 0.533 | NaN | NaN | 0.025 | 5.053 | 0.000 | 19.495 | 1.460 | 10.317 | NaN | NaN | 0.054 | 0.589 |
| 1374105_at | 8 | 8 | 0 | 16.725 | 0.867 | 2.276 | 0.158 | 18.688 | NaN | NaN | 0.058 | 5.642 | 0.562 | 17.522 | 3.113 | 19.537 | NaN | NaN | 0.041 | 0.589 |
| 1390021_at | 2 | 5 | 0 | 0.004 | 1.296 | 10.841 | 1.730 | 13.092 | NaN | NaN | 0.060 | 9.352 | 0.014 | 17.692 | 0.196 | 4.739 | NaN | NaN | 0.044 | 0.196 |
| 1372308_at | 0 | 3 | 0 | NaN | NaN | NaN | NaN | 19.965 | NaN | NaN | 0.026 | NaN | NaN | NaN | NaN | 15.488 | NaN | NaN | 0.577 | 1.374 |
| 1373243_at | 5 | 4 | 0 | 3.326 | 0.000 | 13.204 | 0.774 | 0.706 | NaN | NaN | 0.046 | 7.628 | 0.362 | 11.030 | 0.156 | 12.259 | NaN | NaN | 0.031 | 0.982 |
| 1372364_a_at | 4 | 1 | 1 | 0.479 | 4.871 | 8.350 | 5.165 | 0.350 | 6.333 | 6.349 | 0.048 | 1.219 | 12.236 | 8.017 | 18.969 | 0.628 | 10.037 | 3.491 | 0.189 | 0.000 |
| 1371774_at | 4 | 4 | 3 | 5.718 | 19.979 | 0.624 | 14.026 | 1.860 | 20.000 | 2.233 | 0.207 | 7.842 | 0.864 | 3.661 | 5.948 | 2.093 | 5.686 | 3.143 | 0.148 | 0.982 |
| 1373901_at | 4 | 5 | 0 | 8.742 | 0.228 | 12.173 | 0.186 | 13.585 | NaN | NaN | 0.039 | 3.702 | 0.000 | 19.268 | 0.762 | 8.423 | NaN | NaN | 0.047 | 0.393 |
| 1389965_at | 0 | 4 | 1 | NaN | NaN | NaN | NaN | 20.000 | 0.148 | 0.199 | 0.088 | NaN | NaN | NaN | NaN | 15.382 | 0.113 | 0.000 | 0.082 | 0.589 |
| 1386864_at | 2 | 6 | 1 | 0.099 | 0.152 | 4.161 | 4.762 | 19.999 | 16.168 | 5.260 | 0.041 | 0.897 | 3.623 | 1.851 | 15.397 | 11.144 | 14.085 | 5.867 | 0.062 | 0.196 |
| 1374798_at | 6 | 3 | 1 | 3.520 | 9.455 | 4.181 | 10.571 | 1.956 | 19.998 | 5.170 | 0.067 | 7.797 | 19.999 | 6.748 | 4.582 | 1.026 | 19.999 | 3.067 | 0.074 | 0.982 |
| 1371249_at | 2 | 5 | 0 | 19.999 | 2.898 | 0.443 | 0.917 | 20.000 | NaN | NaN | 0.106 | 1.660 | 0.000 | 17.620 | 0.599 | 11.289 | NaN | NaN | 0.032 | 2.160 |
| 1370283_at | 2 | 3 | 0 | 0.001 | 7.525 | 18.852 | 0.941 | 20.000 | NaN | NaN | 0.095 | 0.000 | 2.903 | 17.761 | 6.860 | 20.000 | NaN | NaN | 0.141 | 0.589 |
| 1389587_at | 6 | 5 | 1 | 4.263 | 0.294 | 10.913 | 3.503 | 6.926 | 6.741 | 6.159 | 0.113 | 5.991 | 0.062 | 19.997 | 18.520 | 9.043 | 20.000 | 7.584 | 0.059 | 1.374 |
| 1374752_at | 3 | 1 | 0 | 5.036 | 13.266 | 14.469 | 4.131 | 0.532 | NaN | NaN | 0.246 | 0.899 | 9.805 | 11.172 | 8.110 | 0.569 | NaN | NaN | 0.094 | 0.196 |

## Liver-Muscle

**Table D: Genes that are commonly oscillating in liver and muscle along with the optimized parameters used to fit their expression in the different tissues (Manuscript Equation 6).** Ebox/RRE/Dbox binding elements (n1, n2, n3) resulted from transcription factor binding site analysis at the promoter regions of the respective genes.

| ProbeIDs | Ebox | RRE | Dbox | Liver | | | | | | | | Muscle | | | | | | | | Δφ  (rads) |
| --- | --- | --- | --- | --- | --- | --- | --- | --- | --- | --- | --- | --- | --- | --- | --- | --- | --- | --- | --- | --- |
|  | **n1** | **n2** | **n3** | **b** | **ba** | **cr** | **gr** | **ar** | **f** | **fa** | **d** | **b** | **ba** | **cr** | **gr** | **ar** | **f** | **fa** | **d** |  |
| 1390430_at | 8 | 5 | 2 | 5.348 | 10.000 | 2.500 | 2.163 | 4.538 | 9.065 | 1.588 | 0.233 | 2.260 | 11.041 | 5.956 | 8.435 | 5.819 | 12.903 | 4.032 | 1.000 | 0.196 |
| 1387703_a_at | 3 | 4 | 0 | 19.993 | 0.108 | 1.424 | 0.069 | 20.000 | NaN | NaN | 0.245 | 19.819 | 0.000 | 3.428 | 0.508 | 19.973 | NaN | NaN | 0.204 | 0.000 |
| 1387874_at | 4 | 2 | 2 | 5.846 | 0.324 | 0.598 | 0.146 | 3.899 | 11.999 | 2.883 | 0.898 | 2.138 | 2.269 | 0.578 | 20.000 | 1.668 | 16.554 | 18.265 | 0.555 | 0.000 |
| 1370510_a_at | 2 | 6 | 0 | 5.638 | 11.937 | 15.660 | 2.923 | 6.139 | NaN | NaN | 0.254 | 0.008 | 17.275 | 11.357 | 19.945 | 6.331 | NaN | NaN | 0.207 | 0.000 |
| 1386946_at | 4 | 1 | 1 | 5.227 | 18.199 | 1.120 | 2.267 | 1.222 | 20.000 | 1.190 | 0.173 | 10.490 | 20.000 | 20.000 | 6.095 | 1.706 | 20.000 | 0.160 | 0.244 | 0.393 |
| 1373108_at | 5 | 4 | 0 | 6.175 | 0.127 | 19.961 | 10.935 | 3.658 | NaN | NaN | 0.415 | 16.636 | 1.188 | 15.595 | 0.389 | 0.950 | NaN | NaN | 0.159 | 0.589 |
| 1373866_at | 3 | 2 | 1 | 19.956 | 1.804 | 1.249 | 0.059 | 19.989 | 19.981 | 19.999 | 0.132 | 5.288 | 0.653 | 0.129 | 6.986 | 20.000 | 19.994 | 20.000 | 0.132 | 0.589 |
| 1368303_at | 5 | 2 | 2 | 19.192 | 0.302 | 20.000 | 20.000 | 1.685 | 19.914 | 11.958 | 0.772 | 3.193 | 19.999 | 1.886 | 0.786 | 10.696 | 20.000 | 3.584 | 0.101 | 0.589 |
| 1370816_at | 7 | 2 | 1 | 3.744 | 19.995 | 1.816 | 19.649 | 2.359 | 19.999 | 3.742 | 0.297 | 7.703 | 20.000 | 20.000 | 20.000 | 4.897 | 20.000 | 3.046 | 1.000 | 0.393 |
| 1374636_at | 4 | 4 | 1 | 19.857 | 5.920 | 0.264 | 20.000 | 8.502 | 19.993 | 0.722 | 0.062 | 12.037 | 16.311 | 20.000 | 20.000 | 9.783 | 20.000 | 6.785 | 1.000 | 0.982 |
| 1390199_at | 5 | 4 | 0 | 3.969 | 0.000 | 13.633 | 7.003 | 3.402 | NaN | NaN | 0.163 | 3.355 | 0.578 | 10.935 | 5.893 | 1.356 | NaN | NaN | 0.099 | 0.982 |
| 1398246_s_at | 8 | 7 | 1 | 8.826 | 0.207 | 20.000 | 4.329 | 3.934 | 4.877 | 6.876 | 0.121 | 9.830 | 0.027 | 20.000 | 19.805 | 7.773 | 19.998 | 9.881 | 0.225 | 0.393 |
| 1371953_at | 4 | 5 | 0 | 7.967 | 19.981 | 20.000 | 14.112 | 8.841 | NaN | NaN | 0.171 | 2.096 | 1.332 | 2.642 | 13.237 | 7.338 | NaN | NaN | 0.054 | 0.982 |
| 1388901_at | 8 | 4 | 0 | 6.550 | 0.291 | 18.632 | 4.549 | 12.292 | NaN | NaN | 0.078 | 11.556 | 0.000 | 19.954 | 1.870 | 4.571 | NaN | NaN | 0.081 | 1.767 |
| 1369150_at | 7 | 4 | 2 | 8.349 | 0.000 | 20.000 | 3.072 | 3.404 | 0.002 | 19.703 | 0.163 | 5.387 | 13.692 | 19.995 | 20.000 | 8.613 | 14.001 | 0.785 | 0.227 | 1.571 |
| 1388471_at | 3 | 3 | 0 | 8.112 | 20.000 | 20.000 | 19.682 | 10.995 | NaN | NaN | 0.184 | 13.100 | 1.427 | 16.059 | 15.477 | 6.797 | NaN | NaN | 0.092 | 0.589 |
| 1388395_at | 4 | 6 | 0 | 4.980 | 0.000 | 20.000 | 5.415 | 13.102 | NaN | NaN | 1.000 | 4.265 | 0.002 | 19.882 | 4.760 | 4.794 | NaN | NaN | 0.941 | 0.196 |
| 1399005_at | 3 | 2 | 1 | 1.718 | 8.440 | 9.363 | 2.491 | 1.126 | 16.685 | 0.002 | 0.076 | 4.188 | 0.516 | 3.533 | 13.379 | 1.550 | 19.955 | 2.239 | 0.295 | 0.785 |
| 1387294_at | 12 | 5 | 1 | 4.198 | 19.578 | 19.988 | 19.991 | 5.929 | 19.990 | 3.551 | 0.172 | 2.761 | 0.046 | 19.996 | 20.000 | 2.438 | 0.088 | 0.033 | 0.105 | 0.982 |
| 1370019_at | 6 | 3 | 0 | 2.667 | 0.000 | 16.487 | 1.318 | 3.867 | NaN | NaN | 0.043 | 2.880 | 0.001 | 9.970 | 2.812 | 8.676 | NaN | NaN | 0.139 | 1.571 |
| 1368488_at | 1 | 2 | 2 | 19.824 | 0.062 | 19.985 | 15.050 | 5.600 | 1.118 | 7.702 | 0.289 | 19.639 | 2.230 | 3.030 | 19.628 | 2.642 | 2.433 | 4.059 | 0.986 | 0.000 |
| 1370209_at | 7 | 2 | 1 | 19.935 | 13.395 | 10.796 | 1.458 | 0.043 | 20.000 | 9.262 | 1.000 | 10.968 | 20.000 | 20.000 | 1.776 | 0.358 | 20.000 | 4.150 | 1.000 | 0.982 |
| 1388686_at | 2 | 0 | 1 | 7.968 | 0.409 | 20.000 | 18.611 | NaN | 19.570 | 9.266 | 0.176 | 0.000 | 0.291 | 19.327 | 16.714 | NaN | 3.090 | 5.957 | 0.073 | 2.160 |
| 1369973_at | 0 | 6 | 0 | NaN | NaN | NaN | NaN | 19.848 | NaN | NaN | 0.028 | NaN | NaN | NaN | NaN | 19.995 | NaN | NaN | 0.014 | 0.982 |
| 1368549_at | 2 | 2 | 1 | 3.400 | 8.114 | 0.355 | 19.429 | 2.571 | 19.888 | 7.127 | 0.352 | 5.583 | 1.220 | 1.084 | 8.278 | 2.360 | 20.000 | 5.847 | 0.749 | 0.589 |
| 1368249_at | 6 | 2 | 0 | 8.982 | 3.668 | 1.511 | 0.778 | 9.909 | NaN | NaN | 0.042 | 13.100 | 0.550 | 3.180 | 1.541 | 0.037 | NaN | NaN | 0.118 | 1.178 |
| 1388426_at | 3 | 10 | 0 | 12.570 | 0.157 | 20.000 | 2.217 | 20.000 | NaN | NaN | 0.239 | 10.898 | 0.086 | 2.768 | 1.186 | 7.167 | NaN | NaN | 0.151 | 0.982 |
| 1375677_at | 2 | 2 | 0 | 17.807 | 3.767 | 0.250 | 3.285 | 19.260 | NaN | NaN | 0.073 | 4.228 | 14.916 | 19.999 | 1.069 | 19.228 | NaN | NaN | 0.193 | 0.589 |
| 1371583_at | 1 | 5 | 1 | 20.000 | 10.088 | 0.249 | 1.133 | 20.000 | 0.000 | 19.998 | 0.094 | 19.348 | 4.716 | 0.011 | 19.998 | 17.245 | 12.025 | 5.108 | 0.270 | 0.196 |

## Muscle-Adipose

**Table E: Genes that are commonly oscillating in muscle and adipose along with the optimized parameters used to fit their expression in the different tissues (Manuscript Equation 6).** Ebox/RRE/Dbox binding elements (n1, n2, n3) resulted from transcription factor binding site analysis at the promoter regions of the respective genes.

| ProbeIDs | Ebox | RRE | Dbox | Muscle | | | | | | | | Adipose | | | | | | | | Δφ |
| --- | --- | --- | --- | --- | --- | --- | --- | --- | --- | --- | --- | --- | --- | --- | --- | --- | --- | --- | --- | --- |
|  | **n1** | **n2** | **n3** | **b** | **ba** | **cr** | **gr** | **ar** | **f** | **fa** | **d** | **b** | **ba** | **cr** | **gr** | **ar** | **f** | **fa** | **d** |  |
| 1390430_at | 8 | 5 | 2 | 2.260 | 11.041 | 5.956 | 8.435 | 5.819 | 12.903 | 4.032 | 1.000 | 14.566 | 1.514 | 4.121 | 0.664 | 4.349 | 0.148 | 0.254 | 0.219 | 0.000 |
| 1387874_at | 4 | 2 | 2 | 2.138 | 2.269 | 0.578 | 20.000 | 1.668 | 16.554 | 18.265 | 0.555 | 2.441 | 1.720 | 0.639 | 3.573 | 1.217 | 16.191 | 16.738 | 0.951 | 0.196 |
| 1370510_a_at | 2 | 6 | 0 | 0.008 | 17.275 | 11.357 | 19.945 | 6.331 | NaN | NaN | 0.207 | 0.000 | 7.177 | 19.731 | 19.549 | 5.187 | NaN | NaN | 0.220 | 0.196 |
| 1373866_at | 3 | 2 | 1 | 5.288 | 0.653 | 0.129 | 6.986 | 20.000 | 19.994 | 20.000 | 0.132 | 14.314 | 14.011 | 1.268 | 0.329 | 20.000 | 20.000 | 7.240 | 0.044 | 0.589 |
| 1368303_at | 5 | 2 | 2 | 3.193 | 19.999 | 1.886 | 0.786 | 10.696 | 20.000 | 3.584 | 0.101 | 1.239 | 17.455 | 3.817 | 2.542 | 19.950 | 20.000 | 8.176 | 0.175 | 0.000 |
| 1370816_at | 7 | 2 | 1 | 7.703 | 20.000 | 20.000 | 20.000 | 4.897 | 20.000 | 3.046 | 1.000 | 1.379 | 12.569 | 2.641 | 19.996 | 8.755 | 14.918 | 0.840 | 0.773 | 0.196 |
| 1398246_s_at | 8 | 7 | 1 | 9.830 | 0.027 | 20.000 | 19.805 | 7.773 | 19.998 | 9.881 | 0.225 | 15.840 | 0.080 | 19.990 | 19.994 | 6.146 | 19.737 | 9.637 | 0.324 | 0.196 |
| 1388901_at | 8 | 4 | 0 | 11.556 | 0.000 | 19.954 | 1.870 | 4.571 | NaN | NaN | 0.081 | 4.127 | 0.008 | 6.016 | 2.030 | 19.492 | NaN | NaN | 0.124 | 1.178 |
| 1368488_at | 1 | 2 | 2 | 19.639 | 2.230 | 3.030 | 19.628 | 2.642 | 2.433 | 4.059 | 0.986 | 6.886 | 19.998 | 2.741 | 1.774 | 1.187 | 2.103 | 9.663 | 0.373 | 0.000 |
| 1370209_at | 7 | 2 | 1 | 10.968 | 20.000 | 20.000 | 1.776 | 0.358 | 20.000 | 4.150 | 1.000 | 10.597 | 2.724 | 2.301 | 2.492 | 0.080 | 5.856 | 19.974 | 0.068 | 0.589 |
| 1375677_at | 2 | 2 | 0 | 4.228 | 14.916 | 19.999 | 1.069 | 19.228 | NaN | NaN | 0.193 | 15.990 | 2.530 | 0.471 | 0.615 | 18.438 | NaN | NaN | 0.076 | 0.393 |
| 1371583_at | 1 | 5 | 1 | 19.348 | 4.716 | 0.011 | 19.998 | 17.245 | 12.025 | 5.108 | 0.270 | 19.351 | 9.247 | 0.702 | 4.248 | 19.043 | 6.369 | 14.940 | 1.000 | 0.000 |
| 1367850_at | 8 | 7 | 1 | 18.823 | 0.129 | 19.953 | 19.986 | 6.874 | 19.985 | 6.410 | 0.682 | 17.620 | 0.070 | 20.000 | 19.996 | 5.809 | 19.992 | 10.240 | 0.284 | 0.196 |
| 1370847_at | 7 | 5 | 0 | 12.690 | 0.000 | 19.607 | 7.445 | 3.349 | NaN | NaN | 0.064 | 8.650 | 0.000 | 19.989 | 0.558 | 0.154 | NaN | NaN | 0.061 | 0.196 |
| 1368025_at | 4 | 0 | 1 | 19.984 | 3.236 | 0.229 | 0.522 | NaN | 19.999 | 19.239 | 0.085 | 5.392 | 0.093 | 20.000 | 7.845 | NaN | 0.000 | 20.000 | 0.253 | 1.178 |
| 1372091_at | 2 | 2 | 2 | 20.000 | 5.998 | 1.035 | 1.439 | 0.682 | 6.630 | 14.246 | 0.659 | 17.189 | 20.000 | 7.504 | 1.466 | 1.186 | 1.947 | 8.976 | 0.620 | 1.178 |
| 1398365_at | 2 | 3 | 0 | 0.171 | 3.077 | 13.588 | 1.673 | 14.197 | NaN | NaN | 0.092 | 0.000 | 3.375 | 16.343 | 1.283 | 19.995 | NaN | NaN | 0.202 | 0.393 |

## Muscle-Lung

**Table F: Genes that are commonly oscillating in muscle and lung along with the optimized parameters used to fit their expression in the different tissues (Manuscript Equation 6).** Ebox/RRE/Dbox binding elements (n1, n2, n3) resulted from transcription factor binding site analysis at the promoter regions of the respective genes.

| ProbeIDs | Ebox | RRE | Dbox | Muscle | | | | | | | | Lung | | | | | | | | Δφ  (rads) |
| --- | --- | --- | --- | --- | --- | --- | --- | --- | --- | --- | --- | --- | --- | --- | --- | --- | --- | --- | --- | --- |
|  | **n1** | **n2** | **n3** | **b** | **ba** | **cr** | **gr** | **ar** | **f** | **fa** | **d** | **b** | **ba** | **cr** | **gr** | **ar** | **f** | **fa** | **d** |  |
| 1390430_at | 8 | 5 | 2 | 2.260 | 11.041 | 5.956 | 8.435 | 5.819 | 12.903 | 4.032 | 1.000 | 2.218 | 9.404 | 4.535 | 3.939 | 6.209 | 5.736 | 3.301 | 0.990 | 0.000 |
| 1387703_a_at | 3 | 4 | 0 | 19.819 | 0.000 | 3.428 | 0.508 | 19.973 | NaN | NaN | 0.204 | 19.962 | 0.541 | 1.456 | 0.117 | 20.000 | NaN | NaN | 0.120 | 0.000 |
| 1387874_at | 4 | 2 | 2 | 2.138 | 2.269 | 0.578 | 20.000 | 1.668 | 16.554 | 18.265 | 0.555 | 1.169 | 3.034 | 0.529 | 14.063 | 9.026 | 9.809 | 14.403 | 0.998 | 0.196 |
| 1370510_a_at | 2 | 6 | 0 | 0.008 | 17.275 | 11.357 | 19.945 | 6.331 | NaN | NaN | 0.207 | 0.003 | 17.494 | 16.478 | 6.523 | 3.972 | NaN | NaN | 0.214 | 0.196 |
| 1386946_at | 4 | 1 | 1 | 10.490 | 20.000 | 20.000 | 6.095 | 1.706 | 20.000 | 0.160 | 0.244 | 2.461 | 19.750 | 4.523 | 6.035 | 0.400 | 12.740 | 1.903 | 1.000 | 0.196 |
| 1373866_at | 3 | 2 | 1 | 5.288 | 0.653 | 0.129 | 6.986 | 20.000 | 19.994 | 20.000 | 0.132 | 17.404 | 2.976 | 0.174 | 0.687 | 19.926 | 20.000 | 16.150 | 0.040 | 0.589 |
| 1368303_at | 5 | 2 | 2 | 3.193 | 19.999 | 1.886 | 0.786 | 10.696 | 20.000 | 3.584 | 0.101 | 13.695 | 0.940 | 1.241 | 0.058 | 19.999 | 13.672 | 2.021 | 0.132 | 0.196 |
| 1370816_at | 7 | 2 | 1 | 7.703 | 20.000 | 20.000 | 20.000 | 4.897 | 20.000 | 3.046 | 1.000 | 1.868 | 0.807 | 2.353 | 4.165 | 6.524 | 1.763 | 19.970 | 1.000 | 0.196 |
| 1371953_at | 4 | 5 | 0 | 2.096 | 1.332 | 2.642 | 13.237 | 7.338 | NaN | NaN | 0.054 | 2.637 | 1.092 | 7.817 | 3.756 | 3.259 | NaN | NaN | 0.044 | 0.785 |
| 1388901_at | 8 | 4 | 0 | 11.556 | 0.000 | 19.954 | 1.870 | 4.571 | NaN | NaN | 0.081 | 13.909 | 1.764 | 1.694 | 0.258 | 17.953 | NaN | NaN | 0.078 | 1.571 |
| 1388395_at | 4 | 6 | 0 | 4.265 | 0.002 | 19.882 | 4.760 | 4.794 | NaN | NaN | 0.941 | 3.456 | 0.213 | 10.784 | 1.798 | 6.152 | NaN | NaN | 0.192 | 0.196 |
| 1387294_at | 12 | 5 | 1 | 2.761 | 0.046 | 19.996 | 20.000 | 2.438 | 0.088 | 0.033 | 0.105 | 1.165 | 0.125 | 19.996 | 20.000 | 3.201 | 20.000 | 4.312 | 0.058 | 0.589 |
| 1370019_at | 6 | 3 | 0 | 2.880 | 0.001 | 9.970 | 2.812 | 8.676 | NaN | NaN | 0.139 | 9.367 | 1.627 | 8.477 | 0.236 | 13.722 | NaN | NaN | 0.057 | 0.196 |
| 1368488_at | 1 | 2 | 2 | 19.639 | 2.230 | 3.030 | 19.628 | 2.642 | 2.433 | 4.059 | 0.986 | 19.562 | 1.945 | 2.434 | 19.413 | 1.221 | 14.008 | 2.895 | 0.277 | 0.000 |
| 1370209_at | 7 | 2 | 1 | 10.968 | 20.000 | 20.000 | 1.776 | 0.358 | 20.000 | 4.150 | 1.000 | 13.212 | 1.023 | 1.476 | 3.487 | 0.019 | 19.524 | 12.173 | 0.076 | 0.393 |
| 1388686_at | 2 | 0 | 1 | 0.000 | 0.291 | 19.327 | 16.714 | NaN | 3.090 | 5.957 | 0.073 | 0.000 | 0.161 | 19.910 | 18.029 | NaN | 15.558 | 0.000 | 0.050 | 0.196 |
| 1368549_at | 2 | 2 | 1 | 5.583 | 1.220 | 1.084 | 8.278 | 2.360 | 20.000 | 5.847 | 0.749 | 4.310 | 4.534 | 0.799 | 14.010 | 2.067 | 19.379 | 7.168 | 0.140 | 0.589 |
| 1368249_at | 6 | 2 | 0 | 13.100 | 0.550 | 3.180 | 1.541 | 0.037 | NaN | NaN | 0.118 | 13.658 | 1.985 | 0.684 | 0.581 | 12.412 | NaN | NaN | 0.080 | 0.785 |
| 1388426_at | 3 | 10 | 0 | 10.898 | 0.086 | 2.768 | 1.186 | 7.167 | NaN | NaN | 0.151 | 11.671 | 0.536 | 16.378 | 0.429 | 7.043 | NaN | NaN | 0.069 | 0.785 |
| 1371583_at | 1 | 5 | 1 | 19.348 | 4.716 | 0.011 | 19.998 | 17.245 | 12.025 | 5.108 | 0.270 | 18.172 | 2.274 | 0.741 | 19.141 | 18.586 | 0.367 | 19.998 | 0.116 | 0.589 |
| 1370847_at | 7 | 5 | 0 | 12.690 | 0.000 | 19.607 | 7.445 | 3.349 | NaN | NaN | 0.064 | 15.448 | 0.181 | 19.283 | 19.401 | 2.109 | NaN | NaN | 0.211 | 0.196 |
| 1368025_at | 4 | 0 | 1 | 19.984 | 3.236 | 0.229 | 0.522 | NaN | 19.999 | 19.239 | 0.085 | 14.931 | 2.984 | 0.176 | 1.757 | NaN | 11.124 | 11.511 | 0.065 | 0.589 |
| 1367602_at | 6 | 5 | 2 | 10.886 | 20.000 | 20.000 | 19.989 | 11.990 | 20.000 | 2.561 | 1.000 | 7.759 | 20.000 | 20.000 | 20.000 | 8.626 | 19.919 | 1.561 | 0.116 | 0.000 |
| 1374204_at | 9 | 5 | 1 | 5.678 | 0.059 | 19.349 | 17.412 | 0.826 | 19.999 | 19.975 | 0.074 | 1.982 | 7.596 | 18.893 | 3.509 | 1.754 | 16.184 | 0.000 | 0.029 | 0.393 |
| 1373850_at | 5 | 8 | 1 | 2.412 | 0.002 | 8.489 | 3.805 | 19.999 | 19.919 | 4.074 | 0.097 | 5.994 | 0.477 | 7.410 | 16.172 | 17.683 | 19.999 | 8.940 | 0.088 | 1.571 |
| 1387669_a_at | 4 | 2 | 1 | 4.599 | 2.563 | 0.991 | 4.394 | 5.488 | 1.317 | 0.730 | 0.041 | 15.786 | 2.823 | 0.241 | 0.807 | 19.163 | 20.000 | 15.379 | 0.022 | 0.393 |
| 1390391_at | 7 | 1 | 0 | 7.152 | 14.841 | 19.995 | 4.448 | 0.096 | NaN | NaN | 1.000 | 19.915 | 4.820 | 0.530 | 20.000 | 0.027 | NaN | NaN | 0.058 | 0.589 |
| 1373807_at | 1 | 3 | 0 | 12.732 | 0.281 | 19.930 | 9.454 | 18.612 | NaN | NaN | 1.000 | 0.000 | 8.516 | 20.000 | 20.000 | 0.393 | NaN | NaN | 0.029 | 0.982 |
| 1369625_at | 4 | 3 | 0 | 2.184 | 0.833 | 8.891 | 0.866 | 13.490 | NaN | NaN | 0.098 | 19.969 | 0.580 | 20.000 | 0.323 | 0.110 | NaN | NaN | 0.030 | 1.963 |
| 1388583_at | 3 | 4 | 0 | 8.120 | 0.125 | 15.314 | 0.414 | 4.527 | NaN | NaN | 0.043 | 1.832 | 2.300 | 9.637 | 7.726 | 3.210 | NaN | NaN | 0.066 | 1.374 |
| 1371785_at | 5 | 6 | 0 | 8.812 | 0.115 | 19.823 | 0.707 | 4.931 | NaN | NaN | 0.082 | 1.737 | 0.000 | 19.994 | 1.657 | 13.157 | NaN | NaN | 0.065 | 0.196 |
| 1373266_at | 10 | 4 | 0 | 9.753 | 0.187 | 1.749 | 0.290 | 15.395 | NaN | NaN | 0.069 | 20.000 | 0.885 | 3.243 | 0.092 | 20.000 | NaN | NaN | 0.096 | 0.196 |
| 1367568_a_at | 6 | 1 | 1 | 0.967 | 4.840 | 19.999 | 20.000 | 1.215 | 20.000 | 6.842 | 0.348 | 5.440 | 1.438 | 16.723 | 0.147 | 17.794 | 19.998 | 18.494 | 0.023 | 0.000 |
| 1373092_at | 2 | 0 | 0 | 0.438 | 0.266 | 8.229 | 5.986 | NaN | NaN | NaN | 0.290 | 1.655 | 1.282 | 15.914 | 15.742 | NaN | NaN | NaN | 0.150 | 0.785 |
| 1374650_at | 4 | 6 | 0 | 11.989 | 0.000 | 18.730 | 0.099 | 2.204 | NaN | NaN | 0.029 | 2.919 | 0.548 | 8.986 | 2.704 | 4.081 | NaN | NaN | 0.080 | 1.571 |
| 1373595_at | 4 | 2 | 2 | 2.012 | 6.805 | 5.014 | 3.925 | 1.007 | 13.521 | 1.291 | 0.691 | 7.445 | 0.323 | 1.410 | 9.543 | 0.145 | 20.000 | 19.793 | 0.073 | 0.393 |
